# An improved 9 micron thick separator for a 350 Wh/kg lithium metal rechargeable pouch cell

**DOI:** 10.1038/s41467-022-34584-z

**Published:** 2022-11-10

**Authors:** Zhi Chang, Huijun Yang, Anqiang Pan, Ping He, Haoshen Zhou

**Affiliations:** 1 School of Materials Science and Engineering, Key Laboratory of Electronic Packaging and Advanced Functional Materials of Hunan Province, Central South University, Changsha, 410083 Hunan China; 2 Energy Technology Research Institute, National Institute of Advanced Industrial Science and Technology (AIST), 1-1-1, Umezono, Tsukuba, 305-8568 Japan; 3 Center of Energy Storage Materials & Technology, College of Engineering and Applied Sciences, Jiangsu Key Laboratory of Artificial Functional Materials, National Laboratory of Solid State Micro-structures, and Collaborative Innovation Center of Advanced Micro-structures, Nanjing University, Nanjing, 210093 China

**Keywords:** Batteries, Batteries

## Abstract

The use of separators that are thinner than conventional separators (> 20 µm) would improve the energy densities and specific energies of lithium batteries. However, thinner separators increase the risk of internal short circuits from lithium dendrites formed in both lithium-ion and lithium metal batteries. Herein, we grow metal-organic frameworks (MOFs) inside the channels of a polypropylene separator (8 µm thick) using current-driven electrosynthesis, which aggregates the electrolyte in the MOF channels. Compared to unmodified polypropylene separators, the MOF-modified separator (9 µm thick) vastly improves the cycling stability and dendrite resistance of cells assembled with Li metal anodes and carbonate-based electrolytes. As a demonstration, a 354 Wh kg^−1^ pouch cell with a lithium metal anode and LiNi_0.8_Co_0.15_Al_0.05_O_2_ (NCA)-based cathode (N/P = 3.96) is assembled with 9 µm layer of the MOF-modified separator and retains 80% of its capacity after 200 cycles (charged at 75 mA g^−1^, discharged at 100 mA g^−1^) at 25 °C.

## Abstract (web summary)

Thin separators can improve batteries’ energy densities but increase cell shortcircuit risks. Here, the authors report an improved thin metal-organic frameworks separator to improve the dendrite formation resistance and cycling stability of high-voltage lithium battery in carbonate electrolytes.

## Introduction

**Fig. 1:**
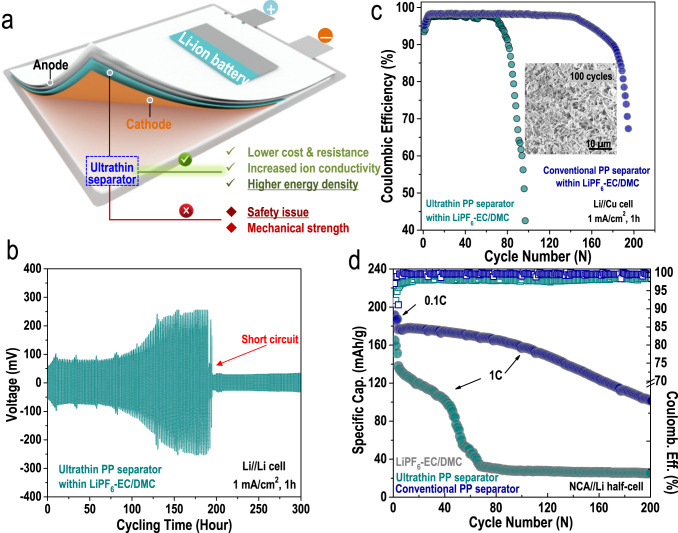
Conventional ultrathin separators (8 µm thickness) are excluded from use in lithium-metal batteries. **a** Schematic representation of the advantages and disadvantages of a conventional ultrathin separator in constructing a Li-ion battery system. Li-metal plating/stripping reversibility evaluated by **b** Li||Li symmetric cell and **c** Coulombic efficiency (CE) of a Li | |Cu half-cell assembled with an ultrathin separator (8 µm thickness) and conventional separator (20 µm thickness) in typical 1 mol/L LiPF_6_-EC-DMC carbonate-based electrolyte: 1 mA/cm^2^, 1 h. **d** Cycling performance of a LiNi_0.8_Co_0.15_Al_0.05_O||Li (NCA||Li) half-cells assembled with an ultrathin separator (8 µm thickness) and a conventional separator (20 µm thickness) in a typical 1 mol/L LiPF_6_-EC-DMC carbonate-based electrolyte at a 1C current rate (0.1C for the first three cycles).

The rapid development of various lighter, thinner and smaller portable electronic devices or even electric vehicles (EVs) calls for batteries with high energy densities but thin volumes^1,2^. Using an ultrathin separator can effectively reduce the overall weights and volumes of batteries^3^. Therefore, ultrathin separators (<10 µm) offer remarkable advantages in gravimetric/volumetric density over conventional separators (>20 µm), which are thicker than the ultrathin separators, not to mention the relatively low costs of the ultrathin separators. Unfortunately, as the thickness is decreased, it is much easier for dendrites to penetrate the ultrathin separators; thus, typical lithium-ion batteries (LIBs, with graphite as the anode) assembled with ultrathin separators tend to suffer from limited cell lifespans and fast capacity decays (Fig. 1a). Moreover, a large drop in the heat resistance and electrolyte absorption abilities caused by the ultrathin separator also contributes to inferior cell performance^4^. Even worse, when an ultrathin separator was used, readily occurring battery short circuits even induced catastrophic battery explosions^5^.

Since lithium metal anodes are more reactive than graphite, ultrathin separators were generally excluded from use in lithium-metal batteries (LMBs); the inherent lithium dendrites can easily penetrate through the ultrathin separator and lead to much faster cell failure and more severe safety hazards^6–8^. For example, as shown in Fig. 1b, the performance of a Li||Li symmetric cell assembled with a commercially available ultrathin separator (8 µm) was only sustained for 170 h (apparent voltage polarization after 100 h), after which a sudden short circuit occurred. After assembly with a commercially available ultrathin separator (8 µm), the Li||Cu half-cell demonstrated in Fig. 1c (dark green curve) also exhibited low coulombic efficiency (CE, 97.7%) and short cycling life (<100 cycles), while the LiNi_0.8_Co_0.15_Al_0.05_O||Li half-cell (NCA||Li, at 1C current rate) in Fig. 1d (dark green curve) exhibited very fast capacity decay. For cells assembled with conventional PP separators (20 µm), both the cycling lives of the Li||Cu half-cells (Fig. 1c, dark blue curve) and the capacities of the NCA||Li half-cells (Fig. 1d, dark blue curve) apparently improved. Numerous lithium dendrites were clearly observed on the surface of lithium harvested from the cycled Li||Cu half-cell assembled with an ultrathin PP separator, as shown in Fig. 1c. These results together verified why commercially available ultrathin separators have not been employed in fabricating LMBs.

To overcome the aforementioned challenging issue of commercially available ultrathin separators that cannot be used in LMBs (especially LMBs producing high energy), separators exhibiting small thickness but excellent toughness to prevent piercing with dendritic lithium are highly desirable. In addition, the thin separators should be lightweight and have excellent toughness (to suppress piercing by the dendritic lithium and prevent battery short-circuiting), and a desirable separator should also exhibit proper electrolyte absorption ability^9,10^. Due to their special subnanoscale structures, metal-organic frameworks (MOFs) have been frequently studied for application in LIBs/LMBs during the past several years^11–21^. Their porous structure was reported to accommodate liquid electrolytes, while the subnanoscale channels/windows facilitated uniform deposition of lithium ions on the lithium-metal surface, thus eliminating the growth of dendritic lithium and the possibility of short circuits^11,14,15^. Combining an ultrathin separator with a MOF offers considerable potential for solving the inherent drawbacks of the commercially available ultrathin separators demonstrated in Fig. 1. Theoretically speaking, the target MOF constructed on the surface of an ultrathin separator should be crack-free, light, and thin. However, it has been a long-lasting challenge to prepare uniform and crack-free MOF films with controllable thickness on the surface of flexible porous membranes, especially on the surfaces of ultrathin porous membranes^10^.

In this work, crack-free ZIF-8 MOF was grown in situ inside the channels/voids of a commercially available ultrathin separator (8 µm) serving as the host matrix via current-driven electrosynthesis. Inspiringly, the resulting improved ultrathin MOF-based separator was only 9 µm in thickness and exhibited almost the same weight as a pristine commercially available ultrathin separator host matrix (0.82 mg/cm^2^ vs. 0.80 mg/cm^2^). More interestingly, during use in a current-driven process within a coin-cell, the crack-free ultrathin MOF-based separator contained only a trace amount of liquid electrolyte (3.1 mg/cm^2^ in this work vs. 32.4 mg/cm^2^ added into typical cells), which generated an aggregative configuration (compared with a typical diluent electrolyte) inside the channels of the MOF. Benefiting from the use of the improved ultrathin and light MOF-based separator, the Li||Cu half-cell assembled with two times predeposited Li electrode delivered a high average coulombic efficiency (CE) of 99.7% over 1800 h within a typical carbonate electrolyte (1 mol/L LiPF_6_-EC/DMC, which was originally incompatible with lithium-metal). In addition, the NCA||Li full-cell with limited Li (two-fold excess Li calculated based on a NCA cathode) maintained a 176 mAh/g capacity (over 90% of its initial capacity) after even 400 cycles, which far surpassed a NCA||Li full-cell using a typical ultrathin separator (85.5 mAh/g after only 31 cycles, after which the cell failed). Due to the light weights of the separator and electrolyte, we ultimately obtained a 354 Wh/kg lithium-metal-based pouch cell (25.2 mg/cm^2^ NCA mass loading) that sustained 80% of its initial capacity over 200 cycles under a N/P ratio of 3.96 (areal capacity ratio for negative to positive electrodes), and an E/C ratio of 1 g A h^−1^ in a conventional carbonate electrolyte by merely using the improved ultrathin separator.

## Results and discussion

### Design of the improved ultrathin MOF-based separator

**Fig. 2:**
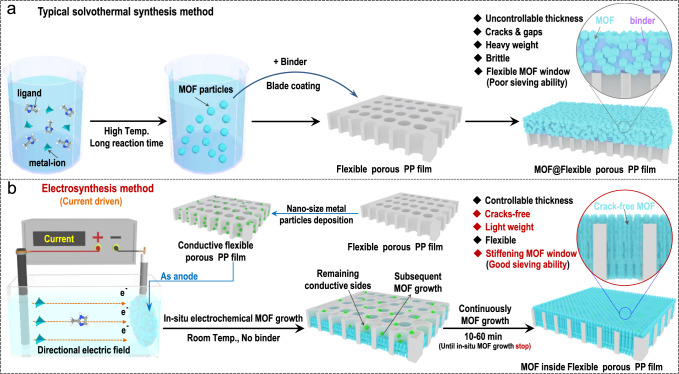
Schematic of the crack-free improved ultrathin MOF-based separator prepared with the current-driven electrosynthetic method. **a** Schematic representation of the typical method for preparing a MOF coated on a flexible porous polypropylene film (PP) by directly blade coating MOF particles prepared through a conventional solvothermal synthetic method and the corresponding inherent drawbacks. **b** Apparatus and conditions used for current-driven electrosynthesis of the crack-free MOF-based separator. Note that the crack-free MOFs were grown in-situ from inside the channels to the surface of the PP separator during the current-driven electrosynthetic process.

The typical way to combine MOFs with flexible porous membranes (for example, polypropylene separators and PP separators) is by directly blade coating MOF particles, which were prepared through a typical solvothermal synthetic method (thoroughly mixed with binder), on the surfaces of flexible porous PP separators, as demonstrated schematically in Fig. 2a. However, several inherent drawbacks were apparent during both MOF synthesis and the subsequent blade coating procedure. A conventional solvothermal synthesis process often requires the use of a sealed, pressurized environment, elevated reaction temperatures (from 100 to 200 °C), and long reaction times (usually 2–8 days). During the blade coating procedure, an additional binder was required to bond MOF particles together. After the MOF particles were successfully coated on the PP surface, however, there were also several long-lasting defects. For example, due to the use of binder, the MOF layer coated on PP showed uncontrollable thickness and was full of cracks and gaps. Correspondingly, the MOF-coated PP separator was generally brittle and had a high overall weight. To avoid the drawbacks of typical solvothermal synthetic methods and subsequent blade coating while preparing the MOF-coated PP separator, a special current-driven electrosynthetic method was used in this work, as shown in Fig. 2b. At room temperature (25 °C) and in the absence of binder, crack-free MOF particles grew in-situ inside the channels of the PP separator and finally filled the channels within only 1 h. Note that the in-situ MOF growth continued until there were no remaining conductive sites, which finally led to a crack-free MOF layer with a controllable thickness. Due to the thin MOF layer, the prepared separator is expected to be light.

**Fig. 3:**
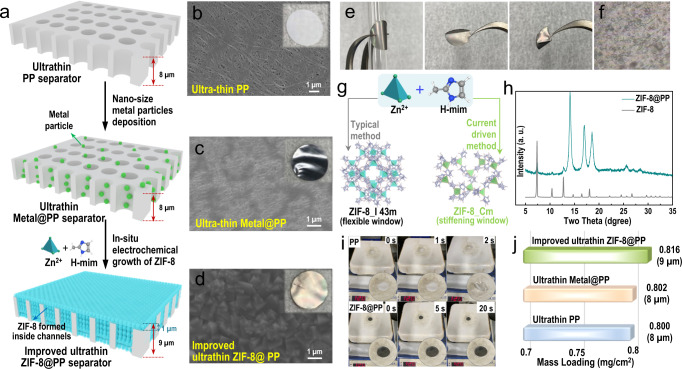
Schematic and characterizations of the crack-free improved ultrathin MOF-based separator. **a** Schematic representation of the preparation process for the improved ultrathin MOF-based ultrathin separator (improved ultrathin ZIF-8@PP separator). Note that the crack-free ZIF-8 MOF was grown in situ from inside the channels to the surface of a conventional ultrathin separator (8 µm) via a current-driven method. Digital photos (insets) and the corresponding SEM images of the **b** conventional ultrathin PP separator, **c** metal particle-coated commercially available ultrathin PP separator and **d** the improved ultrathin ZIF-8@PP separator. **e** Digital photos of the prepared improved ultrathin ZIF-8@PP separator under bending and folding conditions and **f** the corresponding optical microscope photo after folding. **g** Current-driven method used in preparing the improved ultrathin ZIF-8@PP separator. **h** XRD pattern of the improved ultrathin ZIF-8@PP separator. **i** Digital photos of both the commercially available ultrathin PP separator and the improved ultrathin ZIF-8@PP separator at 120 °C. **j** Weight and thickness comparisons for the commercially available ultrathin PP separator and the improved ultrathin ZIF-8@PP separator.

**Fig. 4:**
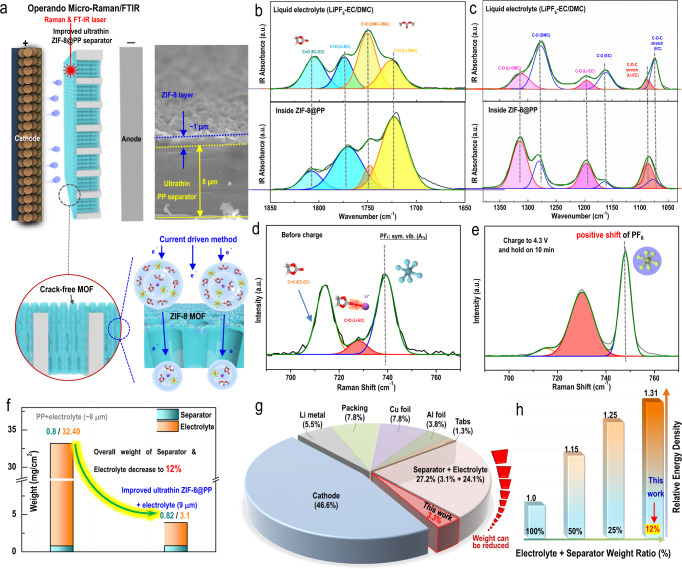
Physicochemical properties of the improved ultrathin ZIF-8@PP separator and the liquid electrolyte inside the channels of the ZIF-8 MOF grown in-situ along the channels of the conventional ultrathin PP separator. **a** Schematic representation of the operando micro-Raman and attenuated total reflection–Fourier transform infrared (ATR-FTIR) techniques employed to characterize the liquid electrolyte confined inside the ZIF-8 MOF grown in-situ and the proposed formation mechanism for the aggregative electrolyte configuration induced by the current-driven method. ATR-FTIR spectra of the typical bulk liquid electrolyte (upper panel) and electrolyte within the ZIF-8 MOF channels (lower panel) for the ranges **b** 1650–1850 cm^–1^ and **c** 1030–1380 cm^–1^. Raman spectra of the electrolyte inside the charged ZIF-8 MOF grown in-situ **d** before and **e** after charging to 4.3 V. **f** Comparison chart for thickness and quality of the conventional ultrathin PP separator and liquid electrolyte generally added in coin-cells and the prepared improved ultrathin ZIF-8@PP separator and the liquid electrolyte it contained for cell fabrication. **g** Pie chart for the weight distributions of all cell components in a pouch cell and the largely decreased weights of the separator and electrolyte used in this work with the prepared ultrathin ZIF-8@PP separator. **h** Improved energy density of a pouch cell assembled with the prepared improved ultrathin ZIF-8@PP separator.

To verify the feasibility of this current-driven electrosynthetic method, an 8 µm commercially available ultrathin PP separator (Fig. 3a, the upper panel and Fig. 3b) was selected as the host matrix for subsequent in-situ growth of a MOF. Generally, an improved ultrathin MOF-based separator was prepared via a fast current-driven method (within one hour), as shown in Fig. 3 and Fig. S1^22,23^. To achieve current-driven MOF growth, the commercially available ultrathin PP separator must be conductive. Conductive metal particles (see the Supplementary Information for details) were deposited on the commercially available ultrathin PP separator (on both the surface and inside the channels), as shown in Fig. 3a (middle panel) and Fig. 3c. The small current (0.7 mA/cm^2^) promoted deprotonation of the H-mim (2-methylimidazole) ligands^24,25^, and then the zinc ions (Zn^2+^) were attracted toward the deprotonated H-mim ligands, which finally led to the formation of the lattice-distorted ZIF-8 MOF (Fig. S1). It is worth noting that the ZIF-8 MOF grew continuously through the porous channels of the commercially available ultrathin PP separator^24^. The obtained ZIF-8@PP separator was 9 µm thick (Fig. 3a, bottom panel; also verified by the SEM image shown in Fig. 4a). Different growth times led to separators with different ZIF-8 MOF coverages (Fig. S2), while a 1-h growth time provided crack-free ZIF-8 MOF (Fig. 3d). The optical interference of the ZIF-8@PP separator indicated that the ZIF-8 MOF was ultrathin and homogeneous (Fig. S3)^22,23^. Formation of the crack-free ZIF-8 MOF was ascribed to the MOF growth mechanism demonstrated in Fig. S4; during the whole growth procedure, all conductive sites (where the conductive metal particles were deposited) were occupied for in-situ growth of the ZIF-8 MOF. The absence of conductive sides and the nonconductive nature of ZIF-8 MOF prevented the ultrathin PP separator host from directly contacting the mother liquid, which in turn prohibited further growth in the thickness of ZIF-8 MOF (Fig. S4). Finally, ZIF-8 MOF formation induced by the fast current-driven synthetic method reported in this work stopped due to the loss of conductive space. The gradually increasing synthetic voltage shown in Fig. S5 also verifies that the separator became nonconductive after complete growth of the nonconductive ZIF-8 MOF. To further verify this, we also used the same fast current-driven synthetic method to prepare ZIF-8 MOF on the surface of a conductive carbon cloth. As shown in Fig. S6, after the pristine conductive carbon cloth fibers were covered by the nonconductive ZIF-8 MOF, the fast current-driven synthetic process stopped (as verified by the high voltage shown in Fig. S6). Moreover, as shown in Fig. S6e, the thickness of the ZIF-8 MOF layer covering the carbon cloth fibers was only ~1 µm (as thick as that of a single layer of MOF particles). This further suggested that once the conductive surface was fully covered by the nonconductive ZIF-8 MOF, the fast current-driven synthetic method just stopped (even when the pristine carbon cloth was conductive). The SEM images shown in Fig. S7 to Fig. S9 indicated the importance of the conductive and porous structure of the host material in successfully preparing the crack-free and uniform ZIF-8 MOF.

The toughness and flexibility of the prepared ZIF-8@PP separator were studied further. As shown in Fig. 3e, the ZIF-8@PP separator was folded and bent without generating any apparent visible cracks (Fig. 3f, optical image of the bent ZIF-8@PP separator), and no fragmentation or detachment of the ZIF-8 MOF was observed, indicating that the ZIF-8@PP separator had good flexibility. The ZIF-8@PP separator preserved its good mechanical properties even after a rigorous tape peeling test (Fig. S10). The excellent toughness and flexibility of the ZIF-8@PP separator benefited from the strong interactions between the ZIF-8 MOF grown in situ and the channels of the commercially available ultrathin PP separator (which were connected by the conductive metal particles predeposited along the channels, as schematically illustrated in Fig. S11)^24,25^. Note that the pore windows of the ZIF-8 MOF grown in situ in this work were stiffened (~3.0 Å) due to the suppressed linker motion induced by lattice distortions during the current-driven method (Fig. 3g, Figs. S1 and S12)^22,23^. The XRD pattern shown in Fig. 3h also suggested successful formation of ZIF-8 MOF within the commercially available ultrathin PP separator. Interestingly, the ZIF-8@PP separator can withstand a high temperature of 120 °C without any structural shrinkage (Fig. 3i, the bottom panel), while the commercially available ultrathin PP separator shrinks within only two seconds (Fig. 3i, the upper panel). In addition, other physicochemical properties (such as electrolyte wettability/uptake, porosity, and thermal stability) of the three separators were measured, and the results are shown in Fig. S13. Clearly, compared with conventional PP and ultrathin PP separators, the improved ultrathin ZIF-8@PP separator exhibited a higher specific surface area, better mechanical stability and much better wettability and thermal stability, further indicating that the improved ultrathin ZIF-8@PP separator was superior to the typical PP separator (ultrathin and conventional PP separator). More inspiringly, the prepared ZIF-8@PP separator was both ultrathin and light (9 µm and 0.816 mg/cm^2^, only slightly thicker and heavier than the commercially available PP separator at 8 µm and 0.800 mg/cm^2^). We thus define it as an improved ultrathin ZIF-8@PP separator.

### Physicochemical properties of the liquid electrolyte confined within the improved ultrathin MOF-based separator

Considering the special structure of the improved ultrathin ZIF-8@PP separator, we think the porous ZIF-8 MOF can act as a liquid electrolyte container and be filled with a certain amount of liquid electrolyte inside the channels of ZIF-8 during battery cycling processes^26^. Operando micro-Raman and attenuated total reflection–Fourier transform infrared (ATR-FTIR) were employed (Fig. 4a, Fig. S14a) to collect detailed information on the liquid electrolyte inside the improved ultrathin ZIF-8@PP separator (ZIF-8 MOF channels) during cycling. The control sample comprised a typical diluent liquid carbonate electrolyte (1 mol/L LiPF_6_-EC/DMC) and was also measured for comparison. As shown in Fig. 4b and c (the upper panel) and Fig. S14b, within a typical diluent electrolyte, the free state of the solvent molecules was the dominant state (the C=O stretching peak (EC-EC) was situated at 1805.2 cm^–1^ and C=O (DMC-DMC) was located at 1750.6 cm^–1^, so the related peak was much stronger than that of C=O (Li-EC) at 1774.2 cm^–1^ and the C=O (Li-DMC) at 1725.9 cm^–1^)^26,27^. For the electrolyte inside the improved ultrathin ZIF-8@PP separator (Fig. 4b, c, the bottom panel), a totally different result was detected; the peaks for coordinated solvent molecules (C=O (Li-EC) and C=O (Li-DMC)) were apparently at much higher frequencies than those of free solvent molecules. This suggested that the electrolyte confined inside the improved ultrathin ZIF-8@PP separator (inside the ZIF-8 MOF channels actually) was more aggregated than typical liquid electrolytes (including dilute and concentrated). Moreover, the blueshift related to Li-EC solvent coordination (to between 1050 and 1450 cm^–1^) also suggested that the liquid electrolyte confined inside the improved ultrathin ZIF-8@PP separator maintained a more aggregated configuration compared with a typical diluent electrolyte^28^. Operando micro-Raman spectroscopy was also used to study changes in configuration for electrolyte confined inside the improved ultrathin ZIF-8@PP separator. Obviously, compared with the Raman peak observed before charging (without potential applied), the Raman signal detected after charging demonstrated clear changes: the peak for Li^+^-bound EC (light red curve in Fig. 4d, e, situated at 728.2 cm^–1^) gradually became the most intense peak, while the EC-EC solvent-related peak (yellow curve in Fig. 4d, e, located at ~714.0 cm^–1^) nearly disappeared. These remarkable peak ratio changes indicated that the liquid electrolyte inside the improved ultrathin ZIF-8@PP separator adopted a more aggregated electrolyte configuration than the typical electrolyte after charging^26^. It is worth noting that the electrolyte confined inside the improved ultrathin ZIF-8@PP separator perfectly maintained its aggregated configuration even after the potential was set from 4.2 V to 2.8 V (Fig. S15, red curve), which suggested that the electrolyte confined inside the improved ultrathin ZIF-8@PP separator, once formed, existed constantly throughout the charging process. More interestingly, the peak related to PF_6_^–^ showed an apparent blueshift (from 739 to 748 cm^–1^) during the process. It was reported that the obvious peak change for PF_6_^–^ was caused by restraint of the symmetrical vibration of the P-F bonds in PF_6_^–^^28^. Since the sizes of the solvated lithium ions within typical 1 M LiPF_6_-EC/DMC (~12 Å)^29^ were much larger than those of the current-driven ZIF-8 channels grown in-situ (~3.0 Å), previously solvated lithium ions needed to dissociate some of the solvent to enter the narrow ZIF-8 channels. This facilitated formation of an aggregated electrolyte inside the ZIF-8 channels. We also thought that the limited spaces of the narrow and stiffening windows generated by in situ current-driven growth of the ZIF-8 contributed to the peak change for PF_6_^–^.

The overall weight of the improved ultrathin ZIF-8@PP separator, which contained liquid electrolyte inside its channels, was also recorded and is demonstrated in Fig. 4f. Clearly, the improved ultrathin ZIF-8@PP separator prepared herein demonstrated a slightly heavier initial weight than the commercially available ultrathin separator (0.82 mg/cm^2^ vs. 0.80 mg/cm^2^); however, the improved ultrathin ZIF-8@PP separator used for cell assembly contained only 3.1 mg/cm^2^ liquid electrolyte inside its channels, which was only 12% of the weight of the liquid electrolyte in a commercially available ultrathin separator commonly used for cell fabrication (32.40 mg/cm^2^, equal to 30 µL liquid electrolyte in each coin cell)^7,8,30^. If this result were used in fabricating lithium-metal-based pouch cells, then the ratio of separator and electrolyte for pouch cells could be significantly reduced from the previous 27.2% to as low as 3.3% (calculated based on Fig. 4f), as demonstrated in Fig. 4g. Benefiting from the reduced weights of the separator and electrolyte, the energy density of the pouch cell assembled with the prepared improved ultrathin ZIF-8@PP separator would improve remarkably. For example, as demonstrated in Fig. 4h, if the weight of the separator and electrolyte was decreased to 50% or 25% of its initial value, then the energy density of the pouch cell would be improved to 1.15 and 1.25 times its pristine value, respectively (Fig. 4h). Encouragingly, as the weight of the prepared improved ultrathin ZIF-8@PP separator (within the liquid electrolyte inside) was 12% of the weight of a commercially available ultrathin PP separator and electrolyte, the energy density of a pouch cell assembled with the prepared improved ultrathin ZIF-8@PP separator would be as high as 1.31 times the value measured for a pouch cell assembled with a typical ultrathin separator and electrolyte. This result was extremely encouraging for constructing high-energy density LMB pouch cells.

### Coin-cell performances based on lithium-metal and the improved ultrathin MOF-based separator within carbonate-based electrolyte

**Fig. 5:**
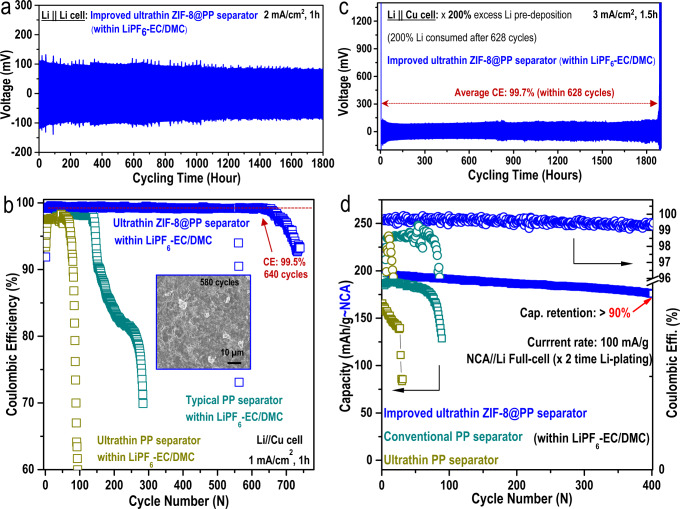
Evaluation of Li-metal plating/stripping reversibility and the cycling performance of an NCA||Li full-cell assembled with the improved ultrathin ZIF-8@PP separator and two-fold excess lithium predeposited. **a** Li||Li symmetric cell assembled with an improved ultrathin ZIF-8@PP separator (9 µm thickness) in a typical 1 mol/L LiPF_6_-EC-DMC carbonate-based electrolyte: 2 mA/cm^2^, 1 h. **b** Coulombic efficiency (CE) of a Li||Cu half-cell assembled with the improved ultrathin ZIF-8@PP separator (9 µm thickness) in a typical 1 mol/L LiPF_6_-EC-DMC carbonate-based electrolyte: 1 mA/cm^2^, 1 h. The CEs of Li||Cu half-cells assembled with a conventional PP separator (20 µm thickness) and a commercially available ultrathin PP separator (8 µm thickness) in a typical 1 mol/L LiPF_6_-EC-DMC carbonate-based electrolyte were also measured for comparison. **c** Specific Li||Cu half-cell: 3 mA/cm^2^, 1.5 h. During the initial two cycles, a two-fold excess of lithium was deposited onto the Cu plate foil. The two-fold excess of lithium was consumed after 628 cycles. **d** Capacity and coulombic efficiency comparisons of NCA||Li full cells (two-fold excess lithium predeposition calculated based on the cathode mass loading) assembled with an improved ultrathin ZIF-8@PP separator, a conventional PP separator (20 µm thickness) and a commercially available ultrathin PP separator (8 µm thickness) in a typical 1 mol/L LiPF_6_-EC-DMC carbonate-based electrolyte.

To further evaluate the use of the improved ultrathin ZIF-8@PP separator with a small amount of liquid electrolyte to fabricate high-performance LMBs, the electrochemical properties of lithium-metal based coin-cells were studied. A Li||Li symmetric cell using the improved ultrathin ZIF-8@PP separator within a typical 1 mol/L LiPF_6_-EC-DMC carbonate-based electrolyte was evaluated first. Obviously, even when measured with a current rate of 2 mA/cm^2^ for 1 hour of plating/stripping time, no apparent voltage polarization or cell short circuiting was observed during a long cycling time of over 1800 h (Fig. 5a). This result was quite encouraging and impressive because it is widely acknowledged that carbonate-based electrolytes are generally incompatible with reactive lithium metal^6,31–37^. This was further verified by the poor electrochemical performance (limited cycling time, fast polarization, and cell short circuit) of the Li||Li symmetric cell using a conventional PP separator (20 µm) and a 1 mol/L LiPF_6_-EC-DMC carbonate-based electrolyte (Fig. S16). The high coulombic efficiency (CE) of the Li||Cu cell is important in constructing a highly efficient lithium metal full-cell. Thus, Li||Cu half-cells assembled with different separators (the prepared improved ultrathin ZIF-8@PP separator, commercially available ultrathin PP separator (8 µm) and a conventional PP separator (20 µm)) were also measured at 1 mA/cm^2^ and for 1 h of plating/stripping (Fig. 5b). Clearly, the Li||Cu half-cell assembled with the commercially available ultrathin PP separator (8 µm) delivered the lowest CE and the fastest CE drop after only 80 cycles (dark yellow cubes in Fig. 5b). When the conventional PP separator (20 µm) was used, the Li||Cu half-cell demonstrated slightly improved CE and cycling stability (Fig. 4b, dark green cubes, CE decay after ~150 cycles). In sharp contrast, the Li||Cu half-cell assembled with the improved ultrathin ZIF-8@PP separator (9 µm) prepared herein delivered the highest CE (99.5%) and the longest cell life (740 cycles), as demonstrated in Fig. 5b (blue cubes). The significantly improved CE suggested high reversibility of the Li-plating/stripping process within the carbonated-based electrolyte when the improved ultrathin ZIF-8@PP separator was used. In addition, the lithium metal harvested from the cycled Li||Cu half-cell assembled with the improved ultrathin ZIF-8@PP separator exhibited a smooth and flat surface, and almost no dendrites were observed (inset in Fig. 5b, 580 cycles), which stood in sharp contrast with the dendrite-rich Li-metal surfaces obtained with the other two Li||Cu half-cells (Fig. S17). Moreover, the corresponding etching XPS results for the Li anodes harvested from the cycled Li||Cu cell using the improved ultrathin ZIF-8@PP separator (shown in Fig. S17d) demonstrated remarkably suppressed electrolyte decomposition-related byproducts. In addition, the NCA||Li half-cell and LiNi_0.8_Co_0.1_Mn_0.1_O||Li (NCM-811||Li) half-cell assembled with the improved ultrathin ZIF-8@PP separator also demonstrated much better electrochemical performance than cells using the conventional 20 µm PP separator (Fig. S18). A Li||Cu half-cell containing 2.0 times the amount of excess Li predeposited onto the bare Cu plate and the improved ultrathin ZIF-8@PP separator (containing a liquid electrolyte) was further investigated. As an exhaustive Li-metal stripping strategy, the stripping curves converted to conventional Li||Cu behavior when the 2.0-fold excess Li was totally consumed. Clearly, the 2.0-fold excess of Li-metal was consumed after 628 cycles, which suggested that a high CE of 99.7% was achieved (Fig. 5c). The high CE was among the highest CEs reported for carbonate-based electrolytes. Encouraged by the high CE obtained with the carbonate-based electrolyte, we also fabricated a high-voltage lithium-metal full cell (NCA||Li full-cell) with the improved ultrathin ZIF-8@PP separator (containing liquid electrolyte) and evaluated its performance. Two other separators were also used to fabricate NCA||Li full cells for comparison (all were tested at a current rate of 100 mA g^−1^). After coupling NCA cathodes and the two-fold excess of predeposited Li-metal (calculated based on the mass loading of the NCA cathode) with the improved ultrathin ZIF-8@PP separator (containing liquid electrolyte), Fig. 5d (the blue curve) shows that the NCA||Li full-cell delivered a high average CE of 99.5% and a very stable cycling performance and finally stabilized at a high discharge capacity of 176 mAh g^−1^ after a long cycling life of 400 cycles (with over 90% capacity retention). In stark contrast, NCA||Li full-cells assembled with a commercially available ultrathin PP separator (8 µm, dark traces in Fig. 5d, with 30 µL of liquid electrolyte added) or a conventional PP separator (20 µm, dark green traces in Fig. 5d, also with 30 µL of liquid electrolyte added) demonstrated very fast capacity decays and much lower CEs. Considering the high NCA cathode mass loading (25 mg/cm^2^), even the 30 µL of electrolyte used was much less than that used in most conventional coin-cells; however, it was much more than that used within the improved ZIF-8@PP separator. In addition, the cycled NCA cathode harvested from the cell using the improved ultrathin ZIF-8@PP separator demonstrated remarkably suppressed formation of electrolyte solvent decomposition-related byproducts compared to the other two NCA cathodes (Fig. S19). Moreover, even when cycled with such a low volume of liquid electrolyte (as exhibited in Fig. 4f), the liquid electrolyte was still be detected inside the depths of the cycled NCA cathode (Fig. S20).

To better understand the important role of the improved ultrathin ZIF-8@PP (with an aggregated liquid electrolyte inside the ZIF-8 MOF channels), an improved ultrathin ZIF-8@PP separator soaked in a typical liquid electrolyte was also employed to fabricate a NCA||Li full cell for comparison (as schematically illustrated in Fig. S21). As shown in Fig. S22, the Li||Cu cell with the ultrathin ZIF-8@PP separator soaked in a typical liquid electrolyte demonstrated a slightly enhanced cycling life and Coulombic efficiency compared to cells using ultrathin PP and conventional PP separators (as shown in Fig. 5b, d) but was still inferior to that of the cell using a crack-free ultrathin ZIF-8@PP separator (with an aggregated electrolyte inside the MOF channels, as shown in Fig. 5b, d, blue curves). When evaluated in the NCA||Li half-cell, the same trend was clearly observed, as shown in Fig. S22b. The cycled lithium anode (Fig. S22c) demonstrated reduced dendrite growth compared to cells using ultrathin PP and conventional PP separators but was still inferior (dendritic Li was still observed) to the cell using the crack-free ultrathin ZIF-8@PP separator (with aggregated electrolyte inside the MOF channels), for which nearly no dendritic lithium was found (inset in Fig. 5b). The SEM image (Fig. S22d and e) and etching XPS results (Fig. S22f and g) for the cycled NCA cathode indicated more severe side reaction-induced byproducts than the cycled NCA cathode harvested from a cell using the improved ultrathin ZIF-8@PP separator (with an aggregated electrolyte inside the MOF channels). However, the CEI layer was slightly thinner than that observed in an NCA cycled within a typical liquid electrolyte (assembled with both ultrathin PP and conventional PP separators, as shown in Fig. S19). These results together indicated that ZIF-8@PP soaked with liquid electrolyte can be used directly to improve the electrochemical properties of batteries to some extent. However, the improvement may not be as large as that seen with the crack-free ultrathin ZIF-8@PP separator (with a liquid electrolyte confined inside the MOF channels). We thought these excellent CE results were closely related to the aggregated electrolyte contained inside the improved ultrathin ZIF-8@PP separator^33^. It is worth noting that the improved ultrathin ZIF-8@PP separator still exhibited a crack-free microstructure after cycling, which showed its excellent stability even after use in various electrochemical processes (Fig. S23).

### Fabrication of a 350 Wh/kg pouch-cell with a carbonate-based electrolyte and the improved ultrathin MOF-based separator

**Fig. 6:**
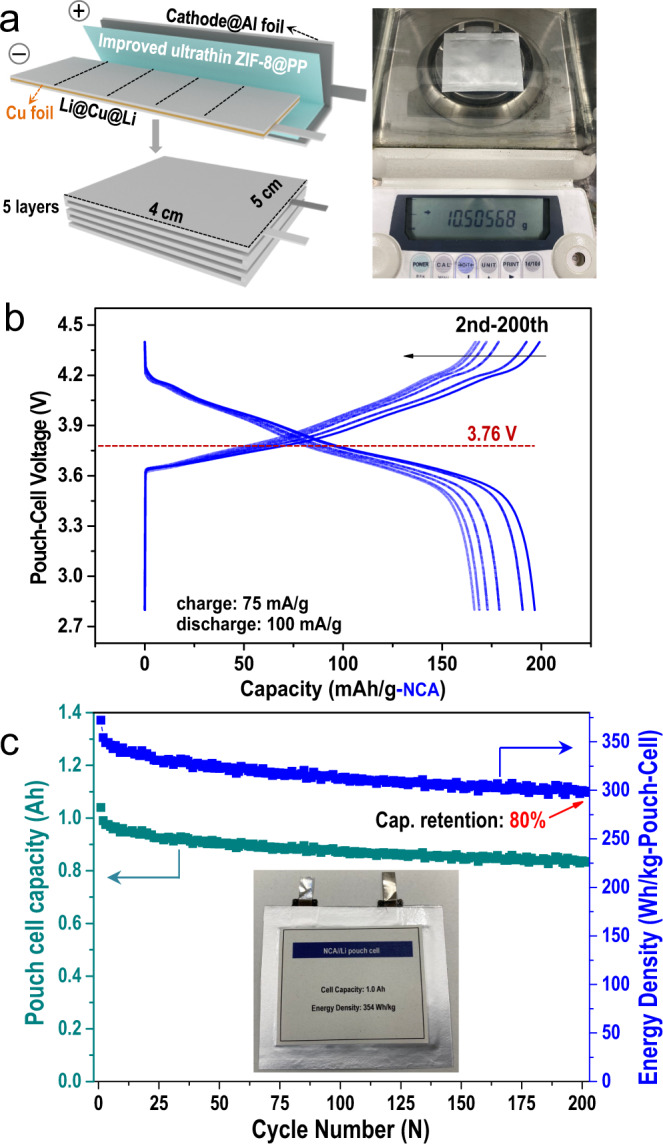
Performance and the corresponding cell parameters for the 350 Wh/kg NCA||Li pouch cell assembled with the improved ultrathin ZIF-8@PP separator in a typical 1 mol/L LiPF_6_-EC/DMC carbonate-based electrolyte. **a** Schematic representation of the NCA||Li pouch-cell packing assembled with the improved ultrathin ZIF-8@PP. **b** Typical galvanostatic charge/discharge curves for pouch-type cell fabricated with a NCA cathode, improved ultrathin ZIF-8@PP separator, and typical 1 mol/L LiPF_6_-EC/DMC carbonate-based electrolyte. **c** Output capacity and energy density of the 350 Wh/kg NCA||Li pouch-cell assembled with the improved ultrathin ZIF-8@PP separator in a typical 1 mol/L LiPF_6_-EC/DMC carbonate-based electrolyte. A digital photo of the pouch cell is shown in the inset for clarity.

The improved ultrathin ZIF-8@PP separator was again employed to fabricate a lithium-metal pouch cell. To improve the energy density of the pouch cell, the mass loading of the NCA cathode material was 25.2 mg/cm^2^ (a cathode mass loading of approximately 31.5 mg/cm^2^), and the pouch cell consisted of five cathode layers and five anode layers (each layer was 4 × 5 cm^2^), as schematically illustrated in Fig. 6a and Fig. S24. The pouch cell exhibited an area capacity of 4.85 mAh/cm^2^. The N/P ratio (the areal capacity ratio of the negative to positive electrodes) was calculated as 3.96. One thing we would like to emphasize is that due to the difficulty in making the pouch cell work normally and efficiently, more liquid electrolyte was added into the pouch cell compared with the coin cell fabricated in Fig. 5d. Even so, the amount of liquid electrolyte used in fabricating the pouch cell was as low as 0.9 g (lead to an extremely low E/C ratio (the ratio of electrolyte weight to cell capacity) of 1 g Ah^−1^). Other parameters for fabricating the pouch cell were also determined, as demonstrated in Table S1. The discharge/charge curves (Fig. 6b), Coulombic efficiency and the slow capacity decay (Fig. S25) suggested the excellent electrochemical performance of the pouch cell. Based on the results shown in Table S1 and 6b, a 354 Wh/kg lithium-metal pouch cell was ultimately obtained, as shown in Fig. 6c. Note that the 354 Wh/kg output energy density was calculated based on the entire weight of the pouch cell (the inset shows a digital photo of the prepared 354 Wh/kg pouch cell). This 354 Wh/kg pouch cell preserved 80% of its initial capacity even after 200 cycles. It is worth noting that the cycled NCA cathode harvested from the pouch cell demonstrated byproducts resulting from electrolyte decomposition (as shown in Fig. S26b, c and Fig. S26 e, f), while the cycled Li anode maintained a dendrite-free surface (Fig. S26d). This can be ascribed to the extra amount of liquid electrolyte added to the side of the improved crack-free ultrathin ZIF-8@PP separator facing the NCA cathode, which further verified the effectiveness of the improved crack-free ultrathin ZIF-8@PP separator in protecting the Li anode (as schematically illustrated in Fig. S26a). To be honest, due to our limited experience with lab-scale pouch-cell fabrication technology, several important parameters (e.g., the ratio and mass loading of active material, Li metal thickness, and electrode architecture) are expected to be improved further, and this will provide considerable improvement in the energy density. Even though there are still some areas for improvement, we still find these results significant because we have obtained these encouraging data solely by using an improved ultrathin ZIF-8@PP separator. With this simple but effective method, R&D on constructing high energy density LMBs is expected to be liberated from complex procedures and use of various expensive concentrated electrolytes composed of lithium salts/additives/cosolvents.

In this work, a current-driven electrosynthetic method was used to prepare an improved ultrathin separator (crack-free ZIF-8@PP separator, 9 µm, 0.82 mg/cm^2^) based on a commercially available ultrathin separator (8 µm, 0.80 mg/cm^2^), which is generally unsuitable for use in LIBs/LMBs. After assembly into a coin-cell within a carbonate-based electrolyte (another current-driven process), the overall weight of the improved ultrathin separator (combined with an aggregated electrolyte inside the MOF channels) was only 12% of that of a cell assembled with a commercially available ultrathin separator (8 µm) and a typical electrolyte (3.1 mg/cm^2^ vs. 32.4 mg/cm^2^). The aggregated electrolyte confined inside the improved ultrathin and light separator contributed to the 99.7% lithium stripping/plating efficiency (1800 hours) for the Li||Cu half-cell assembled with a two-fold excess of Li. Due to the improved ultrathin MOF-based separator, a practical lithium-metal full-cell (with a two-fold excess of Li) with a long cycling life and was achieved with a carbonated-based electrolyte at the coin-cell level, with 176 mAh g^−1^ remaining after 400 cycles (over 90% capacity retention). More practically, we have achieved a high-energy-density lithium-metal pouch cell (31.5 mg/cm^2^ cathode mass loading, equal to 25.2 mg/cm^2^ NCA mass loading, N/P ratio of 3.96, and E/C ratio of 1 g Ah^−1^) with a high pouch-cell level output energy density of 354 Wh/kg and 80% capacity retention after 200 cycles. This improvement at the pouch-cell level could accelerate the development of practical lithium-metal batteries in a much easier and efficient way.

## Methods

All the chemicals employed in this synthesis section were purchased from Wako Pure Chemical Industries Ltd. without additional exception.

### Preparation of ultrathin ZIF-8@PP separator

#### Preparation of mother solution for synthesis of ZIF-8 membranes

A solid mixture of zinc salts (0.55 g zinc acetate) and 0.41 g 2-MIM was dissolved in 50 mL methanol. The molar ratio of 2-MIM/Zn^2+^ in this mother solution was 2:1. The mixed solution was treated by ultrasonication to dissolve all the chemicals.

#### Preparation of ZIF-8 membranes on ultrathin PP separator by fast current-driven synthesis (FCDS)

To make the 8 µm commercially available ultrathin PP separator conductive, Pt particles were pre-deposited on the 8 µm commercially available ultrathin PP separator. By using a Pt sputtering instrument (108Auto, 45 s, under current of 25 mA), certain amount of Pt particles was deposited on the 8 µm commercially available ultrathin PP separator. The 8 µm commercially available ultrathin PP separator with Pt coating were cut into coupons with diameter of 16 mm and fixed between rings of aluminum tape with outer diameter of 18 mm and inner diameter of 12 mm. Then the substrate was immersed into the mother solution prepared as stated above. A current density of 0.7 mA cm^−2^ was conducted for a certain period at room temperature to generate the improved ultrathin ZIF-8 membranes before the membrane was taken out and dried at room temperature^22^. The prepared improved ultrathin ZIF-8@PP separator was then dried under 80 °C for 4 h in drying oven and then followed by vacuumed dried oven at 100 °C overnight to activate the MOF film (extrude possible solvents or water molecules inside the ZIF-8 channels)^14,38^. The prepared activated improved ultrathin ZIF-8@PP separator was then cut into small plates (16 mm in diameter) and re-activated under vacuum at 100 °C overnight before transferred into glove box for further usage. The improved ultrathin ZIF-8@PP separator for pouch-cell fabrication was prepared follow the same procedure except large size commercially available ultrathin PP separator was used (10 × 20 cm^2^ in size, surrounded by aluminum tape).

### Preparation of improved ultrathin ZIF-8@PP separator with liquid electrolyte confined inside

In this work, we selected commercially available 1 mol/L LiFP_6_-EC/DMC (the ratio of EC/DMC is 1:1) carbonated-based electrolyte as the basic electrolyte. To prepare the improved ultrathin ZIF-8@PP separator with liquid electrolyte confined inside, we harvested the cycled improved ultrathin ZIF-8@PP separator from the Li||Li cells after cycled for 10 cycles and used it to fabricate various batteries in this work. The large size (10 × 20 cm^2^ in size) improved ultrathin ZIF-8@PP separator for NCA ||Li pouch-cell was obtained by the similar procedure from a Li||Li pouch-cell after cycled for 10 cycles.

### Electrodes preparation

The obtained LiNi_0.8_Co_0.15_Al_0.05_O_2_ (defined as NCA, purchased from Tianjin Lishen Battery Joint-Stock Co., LTD.); LiNi_0.8_Co_0.1_Mn_0.1_O_2_ (defined as NCM-811, provided by Prof An-Min Cao from Chinese Academy of Sciences (CAS), prepared using methods reported previously)^39^, and lithium foil (Lion Chemical Industry Co., Ltd.) were employed as electrode materials. Generally, 1.0 g electrode powders mixed with carbon black and polyvinylidene fluoride (PVDF, Du Pont-Mitsui Fluorochemicals Co. Ltd.) powder in a ratio of 8:1:1 and then directly stirring for 4 h to get a viscous solution. The obtained slurry was then homogeneously coated onto Al foil current collector by a scraper. After tiny pressing procedure, the active materials-loaded Al foil was vacuum dried at 110 °C overnight. After that, the dried cathodes were under a further calendering process (Hohsen Corp.), the calendering machine has rollers with a diameter of 600 mm and a width of 400 mm that can be tempered up to 70 °C. The maximum possible line load is 2000 N/mm, while the maximum web speed is 30 m/min. After the calendering procedure, the cathodes were sent for another vacuum drying at 110 °C overnight. The mass loading of the NCA/NCM-811 cathode materials was about 25.2 mg/cm^2^. Part of the obtained NCA/NCM-811 cathode was cut into final electrode plates (11 mm in diameter) for coin-cell fabrications, while the rest of the obtained NCA cathode was cut into a rectangle (10 × 20 cm^2^ in size) for pouch-cell fabrications. It is worth noting that for the NCA//Li and NCM-811||Li half-cell, thick unlimited lithium metal (400 µm Li) was used as anode, while for the NCA||Li full-cell, thin limited lithium metal (two times excess lithium pre-deposition on copper foil, calculated based on the cathode mass loading) was used as anode.

### Cell assembly and electrochemical measurements

CR2032 coin cells were assembled in an argon-filled glove box, in which both the moisture and oxygen contents were controlled to be less than 1 ppm. The obtained improved ultrathin ZIF-8@PP separator with liquid electrolyte confined inside was used for cell assembling. For comparisons, cells using typical liquid electrolyte carbonate electrolytes (1 mol/L LiFP_6_-EC/DMC, 30 µL, equals to 32.4 mg/cm^2^ demonstrated in Fig. 3f) were also assembled accompanied with the conventional 20 µm PP separator and 8 µm commercially available ultrathin PP separator. In addition, improved ultrathin ZIF-8@PP separator immersed into typical liquid electrolyte/soaked typical liquid electrolyte was also employed for comparison. It is worth noting that for NCA||Li pouch-cell assembling, additional dose (0.9 g, lead to an extremely low E/C ratio of 1 g Ah^−1^) of liquid typical electrolyte was further added. Before the pouch-cell stacking process, the upper and lower ends of the separator were bonded with a Kapton tape (purchased from 3 M company) to firmly sealed the upper and lower ends of NCA cathode, which can consequently prevent the electrolyte leaking into the lithium anode. After the pouch-cell stacking process, extra amount of liquid electrolyte was added through the tiny gaps between the separator and cathode. After extra amount of liquid electrolyte was added, the packed component was sent for package to prepare NCA//Li pouch-cell. The NCM-811||Li and NCA||Li coin-cells and NCA||Li pouch-cells were operated with a potential limit between: 2.7–4.4 V in the study. It worth noting that the prepared NCA||Li pouch-cell was measured under external pressure^40^. Enclosure for applying stack pressure, pouch cells are uniaxially constrained in a steel enclosure with an adjustable wall which can be tightened to apply varying uniaxial pressures (the stack pressure was about 10 MPa). Before each electrochemical characterization, the cells were kept on open circuit for 10 h. All the potentials in this study were referenced to Li/Li^+^. The galvanostatic electrochemical measurements were carried out under potential control using the battery tester system HJ1001SD8 (Hokuto Denko) at room temperature (25 °C in a climatic chamber). For the Linear sweep voltammetry (LSV) and EIS tests, the electrochemical experiments were carried out under the control of a potentiostat (Potentiostat/Galvanostat PGSTAT30, Autolab Co. Ltd., Netherlands). The current and potential outputs from the potentiostat were recorded by a multifunction data acquisition module/amplifier (PGSTAT30 Differential Electrometer, Autolab), which was controlled by General Purpose Electrochemical Software (GPES).

### Morphology and structure characterization

#### SEM and XRD characterizations

The morphology of the as-prepared improved ultrathin ZIF-8@PP separator, conventional 20 µm PP separator, 8 µm commercially available ultrathin PP separators, pristine cathodes and cycled cathodes and Li anodes were characterized with scanning electron microscopy (SEM, JEOL JSM-6380LV FE-SEM). X-ray diffraction (XRD) measurements were performed on a Bruker D8 Advanced diffractometer fitted with Cu-Kα X-rays (*λ* = 1.5406 Å) radiation at a scan rate of 0.016°/s. For the pre-treatment procedures: The cycled cells were transferred into an Ar glove box once the electrochemical treatments were finished, and the electrodes were extracted from the cell and placed in a glass bottle. The electrode plates were twice rinsed by dimethoxyethane (DME, Sigma Aldrich, 99%) to wash off the electrolyte salt and the residual solvent, and then evaporated in a vacuum chamber, connected to the glove box, for 12 h. The dried electrode plates were moved back to glove box and placed onto a SEM sample holder. The sample holder was sealed in an airtight container and then transferred into the SEM sample loading chamber. Note that, in order to restrain the exposure time to the ambient, samples (cycled electrode plates) were tightly sealed into a glass bottle (fill with Ar gas), and transferred to the related chambers (SEM) as quickly as possible. Thus, we assumed the morphology and the component of electrode surface would not obviously change for such a short time exposure to the open air.

#### BET, mechanical strength, wettability, DSC, thermal stability characterizations of separators

Pore size distribution was determined and calculated using BET method and non-localized density functional theory model. Gurley value (air permeability) was measured on a device (Gurley, 4110 N) by determining the time (s) needed to pass certain volume of air (100 cm^3^) through the separator (1 in 2) under a given pressure (1 kPa). Mechanical strength was applied on a tensile testing machine (Hua Long LZW-100G). Electrolyte contact angle of the sample was determined using a contact angle goniometer (OCA20, Dataphysics). Wettability was further evaluated by measuring the climbing height of liquid electrolyte on separator surface. DSC was carried out on a thermal analyzer (NETZSCH, STA 409) from 30 to 500 °C at the rate of 10 °C min^−1^ under dry flow of Ar.

#### Spatial resolution Operando-Raman Spectroscopy Characterizations

The Raman spectra were recorded using a JASCO microscope spectrometer (NRS-1000DT). The spectral resolution of the Raman spectra in the study was ca. 1.0 cm^−1^. Typically, the scattering signal in Raman spectrum was weak and hard to be investigated. In this case, in order to obtain strong and clear peaks on the spectra, we took advantage of a shell-isolated nanoparticle-enhanced Raman spectroscopy (SHINERS) technique that evidently enhances the scattering signal^41^. Briefly, Au nanoparticles (NSp) were synthesized with a diameter of 30–40 nm as core by a standard sodium citrate reduction method. Then freshly prepared aqueous solution of 1 mM (3-aminopropyl) trimethoxysilane (APS, Sigma Aldrich) was added to the gold sol under vigorous magnetic stirring for 15 min, followed by the addition of a 0.54 wt% sodium silicate solution (Tokyo Chemical Industry Co., Ltd). Then, the solution was heated to 90 °C under vigorous magnetic stirring for 1 h. The series of steps ensure the formation of an ultra-thin SiO_2_ shell (2–4 nm) without any pinhole. The washed and dried Au@SiO_2_ NSp were re-dispersed in ethyl alcohol. Finally, the obtained Au NSp solution was mixed with liquid electrolyte for Raman experiment. These Raman samples were further dried in vacuum at 80 °C for 18 h before assembled into the cell. Note that the amount of deposited NSp was very small, so that we assumed it would not cause any influence on the electrochemical behaviors.

### FT-IR characterizations

#### Attenuated Total Reflection Fourier-transform infrared (ATR-FTIR) characterizations

ATR-FTIR measurements were carried out on an FT/IR-6200 spectrometer (JASCO Corp.) coupled with Platinum Diamond ATR, which consists of a diamond disc as an internal reflection element. The typical electrolyte and ZIF-8@PP separator (with aggregative electrolyte inside MOF channels) were placed on the ATR crystal, and then the spectrum was recorded.

#### ATR-FTIR characterization for cycled NCA cathode

To verify the NCA cathode can be wetted by liquid electrolyte coming out from the MOF channels, the ATR-FTIR spectra of cycled NCA cathode (without washing by solvent) under different depths after different times of Tape peeling test were collected. By constantly peeling off the surface layer of cycled NCA cathode, the internal sections in deep depths of NCA can be exposed. Then, ATR-FTIR measurement was applied to detect whether there were liquid electrolyte peaks contained inside the deep depth of the cycled NCA cathode.

#### Depth-resolution etching X-ray Photoelectron Spectroscopy (XPS) characterization

XPS measurement was performed using a VG scientific ESCALAB 250 spectrometers with monochromic Al Kα Ka source (1486.6 eV) under ultra-high vacuum. Similarly, to prevent long-time exposure to air environment, the samples (after rinsing and dry) were tightly sealed into an Ar-filled bottle and then soon transferred into XPS chamber as quickly as possible. The XPS was equipped with etching with different depth to analysis the component distribution. The total etching depth was about 100 nm.

## Supplementary information

Supplementary information

## Data Availability

Source data are provided with this paper and are also available from the corresponding authors upon request. Source data are provided with this paper.

## Source data

Source Data
