# Supplementary material for: An improved 9 micron thick separator for a 350 Wh/kg lithium metal rechargeable pouch cell
Source: Nat Commun. 2022 Nov 10;13:6788. doi: 10.1038/s41467-022-34584-z (PMC9649724; doi:10.1038/s41467-022-34584-z)
Supplement: Supplementary file 1 — Supplementary information [file 41467_2022_34584_MOESM1_ESM.pdf]

Supplementary Information for

An improved 9 micron thick separator for a 350 Wh/kg lithium metal rechargeable pouch cell

Authors: Zhi Chang[1,2], Huijun Yang[2], Anqiang Pan[1]*, Ping He[3] and Haoshen Zhou[2,3]*

Affiliations:

[1]School of Materials Science and Engineering, Key Laboratory of Electronic Packaging and Advanced Functional Materials of Hunan Province, Central South University, Changsha, 410083, Hunan, China.

[2]Energy Technology Research Institute, National Institute of Advanced Industrial Science and Technology (AIST), 1-1-1, Umezono, Tsukuba 305-8568, Japan.

[3]Center of Energy Storage Materials & Technology, College of Engineering and Applied Sciences, Jiangsu Key Laboratory of Artificial Functional Materials, National Laboratory of Solid State Micro-structures, and Collaborative Innovation Center of Advanced Micro-structures, Nanjing University, Nanjing 210093, P. R. China.

*Correspondence to: pananqiang@csu.edu.cn (Prof. A. P.), hszhou@nju.edu.cn (Prof. H. Z.).

Supplementary Discussion

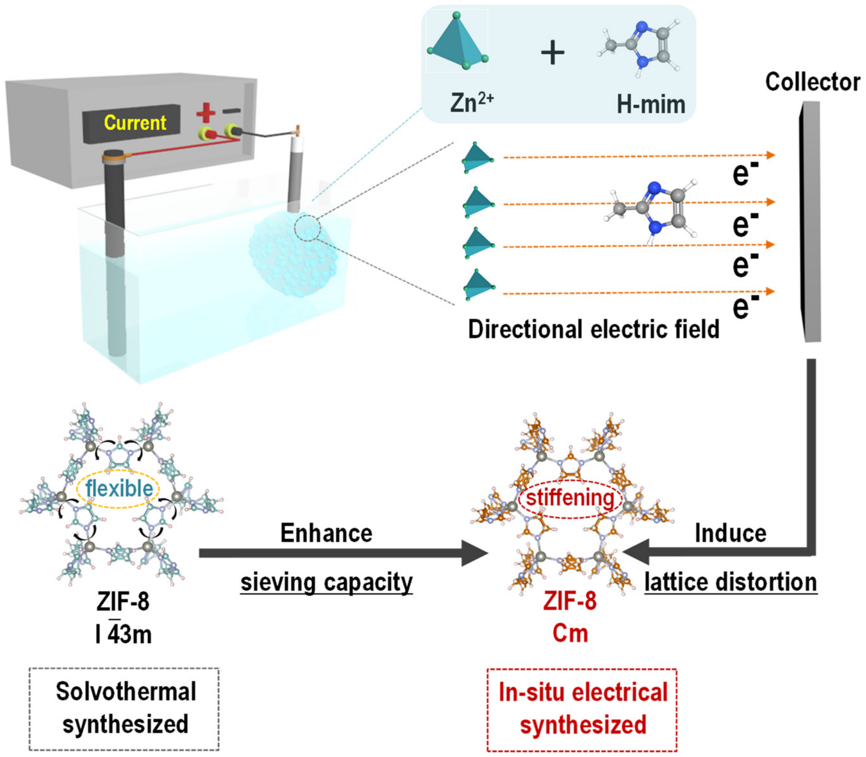

Figure S1. Schematic illustration of preparation of ZIF-8 membrane via fast current driven synthesis (FCDS) on commercially available ultrathin PP separator.

A small direct current (0.7 mA/cm^2) promotes the deprotonation of the linker 2-methylimidazole (mIm) to the imidazolate anions. Simultaneously, Zn^{2+} cations are attracted to the PP substrate serving as a cathode region and meet the deprotonated ligands, thus the formation of ZIF-8 membrane is promoted.

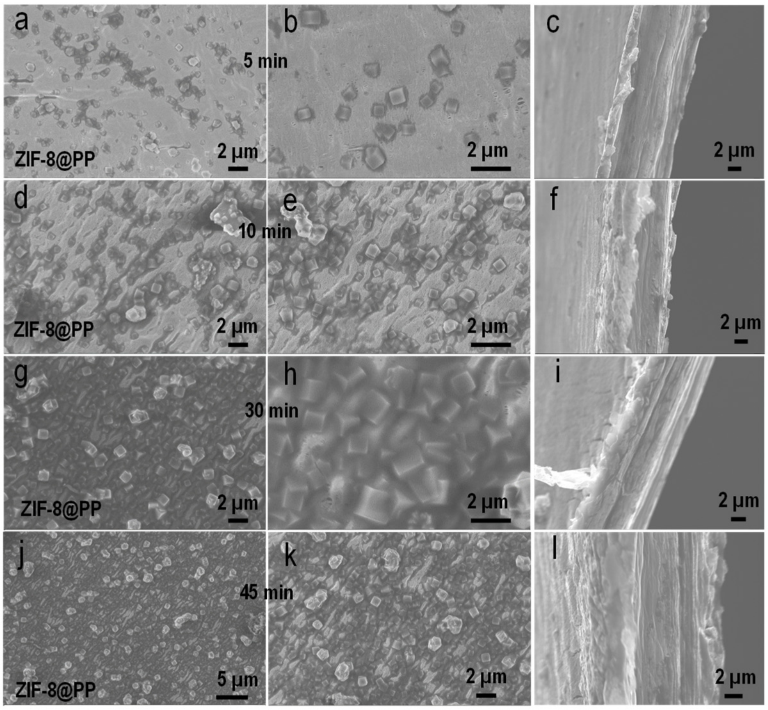

Figure S2. SEM images of the ultrathin ZIF-8@PP separator prepared under different fast current driven synthesis (FCDS) times under (a-c) 5 min, (d-f) 10 min, (g-i) 30 min and (j-l) 45 min.

Different coverage of the ZIF-8 MOF layer was in-situ grown on the surface of ultrathin PP separator. As shown in Figure S2a an S2b, the 5 min sample demonstrated small amount of ZIF-8 grown inside the porous PP separator (with ZIF-8 particles embedded inside the channels/voids of the commercially available ultrathin 8 μm PP separator). When prolonged the FCDS time into 45 min, most of the PP space was covered by the in-situ grown ZIF-8 MOF particles. After 60 min, as shown in Figure 2, a unique crack-free ZIF-8 MOF-based ultrathin and light separator was finally obtained.

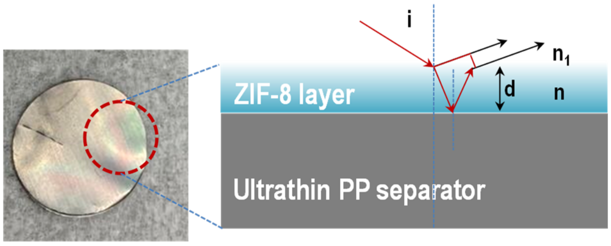

Figure S3. Schematic illustration of the thin-film interference derived from the optical path difference within the ZIF-8 MOF layer.[1]

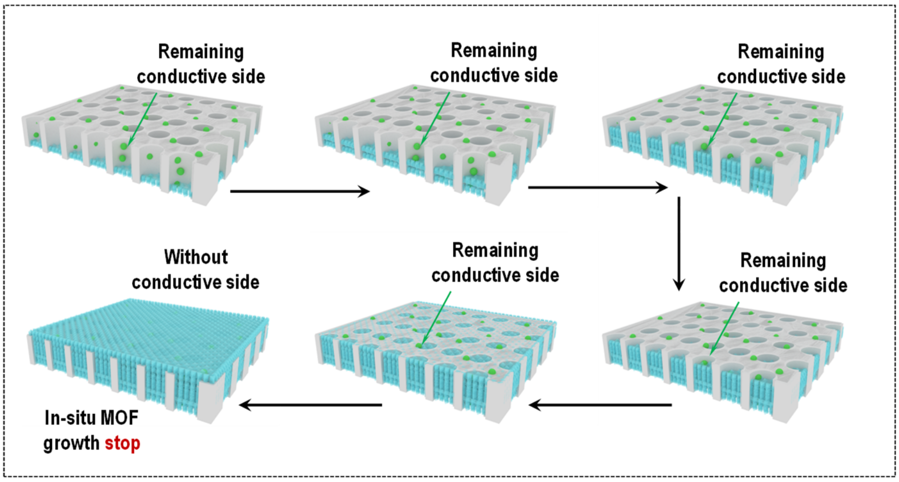

Figure S4. Schematic illustration of the in-situ ZIF-8 MOF growth process for the crack-free improved ultrathin ZIF-8@PP separator.

As shown in Figure S4, if there were remaining conductive sites (metal particles), then the ZIF-8 can in-situ formed on these metal sides. After all the metal sites were covered by non-conducive ZIF-8, the in-situ MOF growth procedure ended.

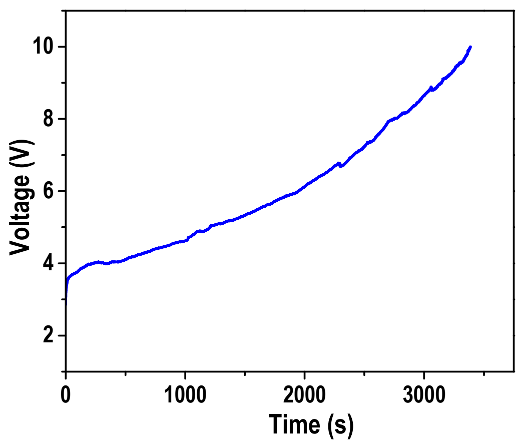

Figure S5. Voltage changes during the fast current-driven synthesis (FCDS) method in fabricating crack-free ultrathin ZIF-8@PP separator.

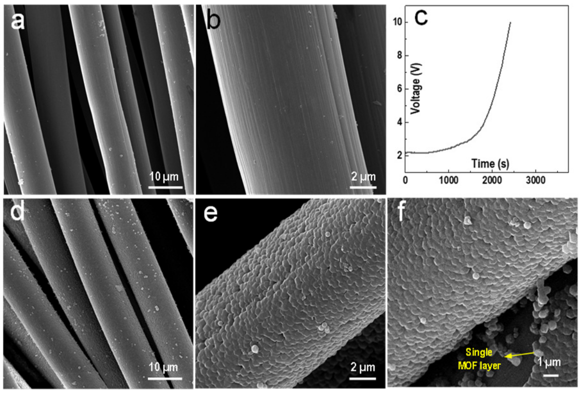

Figure S6. SEM images of (a, b) pristine carbon cloth and (d-f) ZIF-8@carbon cloth using the same fast current-driven synthesis method (FCSD) method as we prepared ZIF-8@PP separator and the corresponding (c) voltage changes during the fabricating process of ZIF-8@carbon cloth.

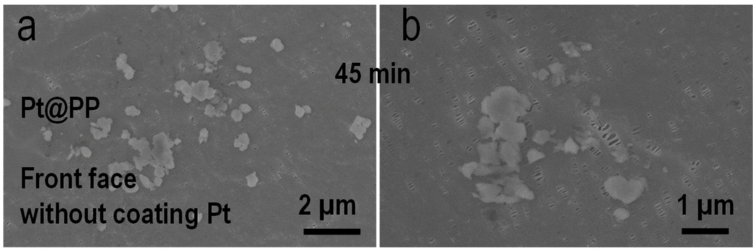

Figure S7. SEM images of the non-conductive side of the Pt@PP separator after fast current driven synthesis (FCDS) process.

The SEM images shown in Figure S7 suggested that the conductive metal side is essential prerequisite for the ZIF-8 MOF to grow inside/on the non-conductive commercially available ultrathin PP separator.

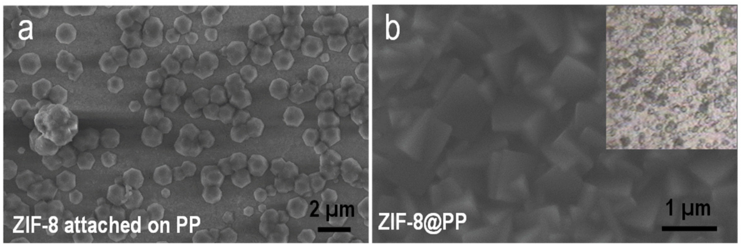

Figure S8. SEM image of (a) ZIF-8 attached on commercially available ultrathin PP and (b) ZIF-8 in-situ grown inside/on commercially available ultrathin PP (the inset is the optical photo of the improved ultrathin ZIF-8@PP).

Clearly, without conductive metal sites, ZIF-8 particles (dodecahedron-like) tend to attached on the surface of commercially available ultrathin PP separator. For sharp contrast, when conductive metal particles were deposited on/inside ultrathin PP separator, crack-free ZIF-8 MOF (square-like) inside/on commercially available ultrathin PP separator scan be obtained.

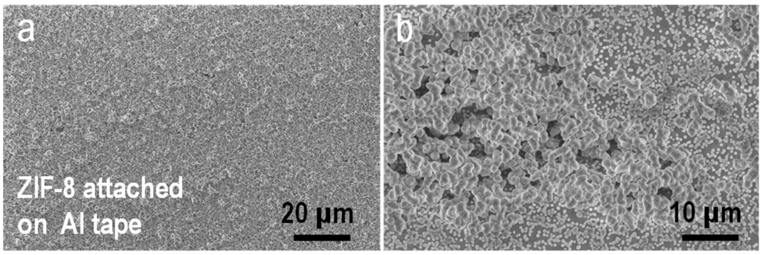

Figure S9. SEM image of ZIF-8 attached on Al foil.

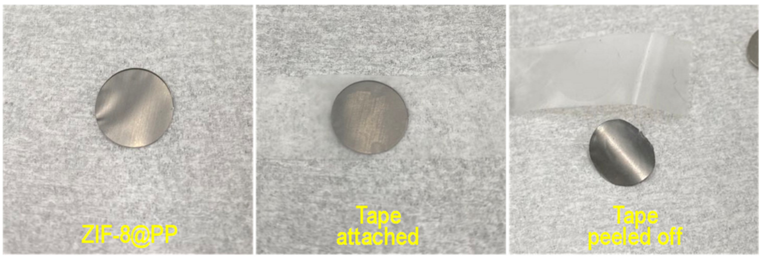

Figure S10. Tape peeling test of the prepared ultrathin ZIF-8@PP separator.

As shown in Figure S10, the in-situ formed ZIF-8 MOF was closely grown one the commercially available ultrathin PP separator after the tape was peeled off. In addition, no any ZIF-8 MOF was observed on the tape, which suggested the excellent mechanical property of the improved ultrathin ZIF-8@PP separator.

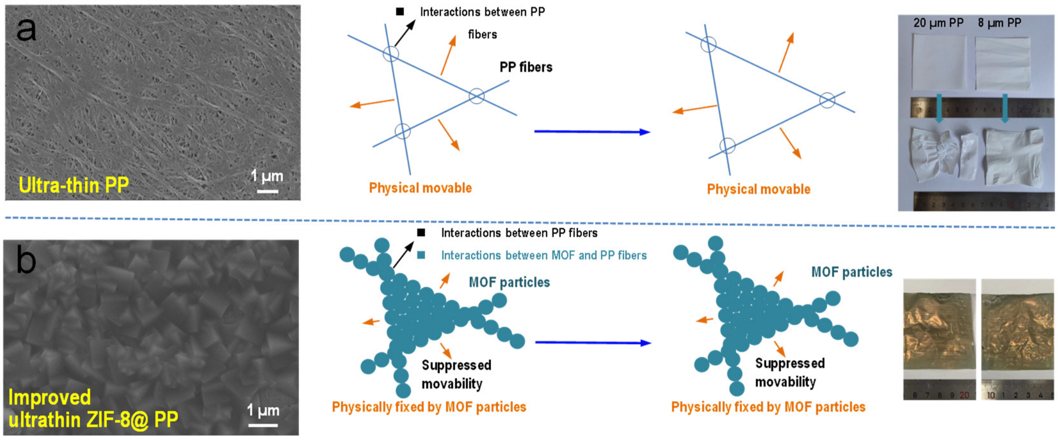

Figure S11. (a) SEM image and the corresponding schematical illustration of conventional PP separator. (b) SEM image and the corresponding schematical illustration of improved ultrathin crack-free ZIF-8@PP separator.

Thanks to the complete filling of MOF particles, the fibers of the ultrathin PP separator which were originally intertwined with each other (as shown in Figure 3b in our original manuscript), were effectively wrapped by the MOF particles. Therefore, those fibers of PP separator which were originally physical movable (interactions between PP fibers, as schematically illustrated in Figure S11a), were finally physically fixed by the MOF particles to some extent (interactions between PP fibers and interactions between MOF particles and PP fibers, as demonstrated in Figure S11b). This enabled the prepared crack-free ultrathin ZIF-8@PP separator additional mechanical strength (as schematically demonstrated in Figure S11b). This is the reason for the prepared ultrathin ZIF-8@PP separator possess good toughness and flexibility.

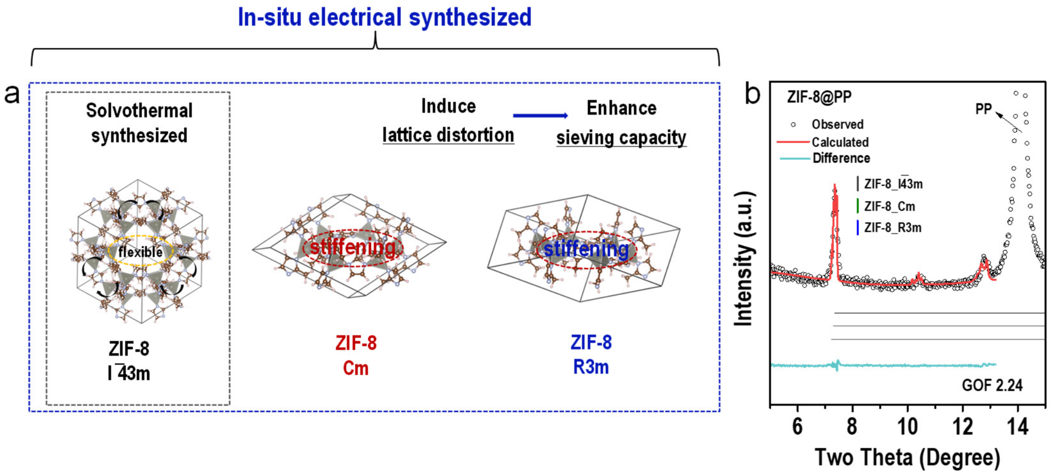

Figure S12. (a) Schematic illustration of the ZIF-8 growth via FCDS in comparison with solvothermal growth. In the solvothermal route, zinc ions and linkers assemble into the normal ZIF-8_I 43m phase. With the local in situ electric field that formed around the support by the current, inborn lattice distortion occurs and the stiff polymorph ZIF-8_Cm and ZIF-8_R3m is formed. (b) Rietveld refinement of the XRD results of FCDS ZIF-8@PP separator.

The different sieving ability of ZIF-8 prepared by typical solvothermal synthesis method and fast current driven synthesis method can be attributed to the different structures of the ZIF-8 prepared by two different methods. The commonly prepared ZIF-8 (composed of flexible ZIF-8_I 43m phase) using typical solvothermal synthesis method do not perform very well sieving ability caused by the inherent lattice flexibility (induced by the rotatable organic linkers), which permits larger molecules to permeate through intracrystalline diffusion and decreases the selectivity/sieving performance. Suppressing the linker mobility is an important route to improve ZIF-8's molecular sieving capability. By introducing an external electric field (induced by the fast current-driven synthesis process), the current-driven synthesized ZIF-8 consisted of a mixture of three polymorphs (ZIF-8_I 43m phase, monoclinic ZIF-8_Cm and trigonal ZIF-8_R3m polymorphs) with a majority of the polymorph ZIF-8_Cm. Rietveld refinement of the XRD results using shows that the ZIF-8 membranes are a mix of three phases with different space groups: Cm, R3m, and I 43m as shown in Figure S12. These three polymorphs of ZIF-8 are also observed when ZIF-8 is exposed to an external electric field. The newly formed monoclinic Cm and trigonal R3m polymorphs exhibited stiffened networks with hindered linker movements, thus improving molecular sieving of ZIF-8.

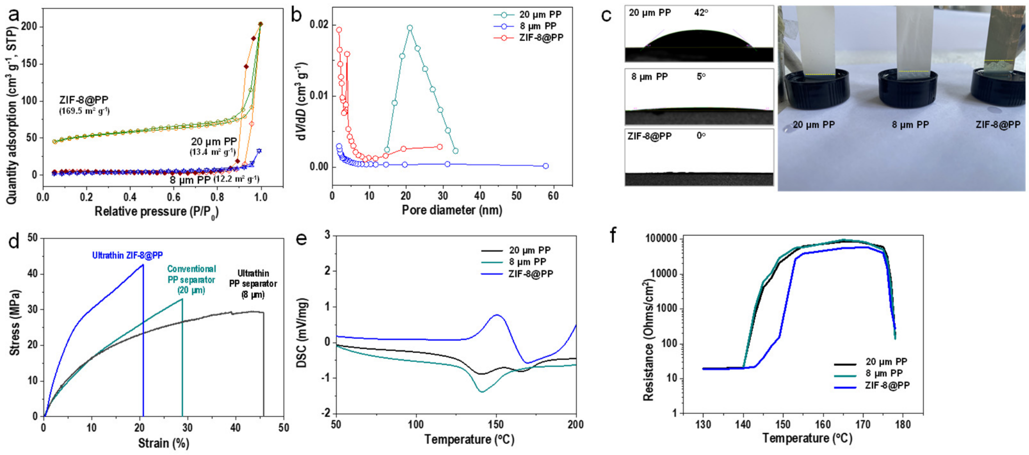

Figure S13. Physicochemical properties of three separators including (a, b) Porosity, (c) wettability, (d) stress-strain profiles of three separators under uniaxial elongation, (e) thermal stability and (f) resistance vs. temperature curves of three separators.

As can be found in Figure S13a, the improved ultrathin ZIF-8@PP separator demonstrated the highest specific surface area (169.5 m^2/g, while the value of ultrathin PP and conventional PP was 13.4 and 12.2 m^2/g, respectively). Due to the existence of ZIF-8 MOF, the improved ultrathin ZIF-8@PP separator exhibited smaller pore sizes distribution. As shown in Figure S13c, the improved ultrathin ZIF-8@PP separator demonstrated much lower contact angle but much faster electrolyte wetting than other two separators. Moreover, as shown in Figure S13d, compared with ultrathin PP (29.2 MPa at a strain of 45.7%) and conventional PP separator (32.9 MPa at a strain of 28.8%), the crack-free ultrathin ZIF-8@PP separator sustained at a much higher yield stress of about 42.7 MPa at a strain of 20.5%, suggesting the much higher breaking strength of the prepared crack-free ultrathin ZIF-8@PP separator. The corresponding data further verified its good mechanical strength. In addition, the shutdown function of three separators was determined by measuring the electrical resistance of the coin cells with the electrolyte-containing separators at elevated temperatures (from 30 to 180 °C) with a heating rate of 5 °C min^{-1}. The sharp rise in impedance around 140 °C was caused by the block off the pores by melted separator, therefore slows down the ionic conduction and cuts off the electrode reactions. With increasing temperature, the impedance of the separators exhibited rapid decline, which indicated that the separators shrink or lose mechanical integrity and can no longer separate the electrodes. The wider temperature window of the improved ultrathin ZIF-8@PP separator than the commercial two separators can be explained by the improved thermal dimensional stability as shown in Figure S13e and f, the improved ultrathin ZIF-8@PP separator also demonstrated higher thermal closure temperature. These data together indicated the improved ultrathin ZIF-8@PP separator was superb than typical PP separator (ultrathin and conventional PP separator).

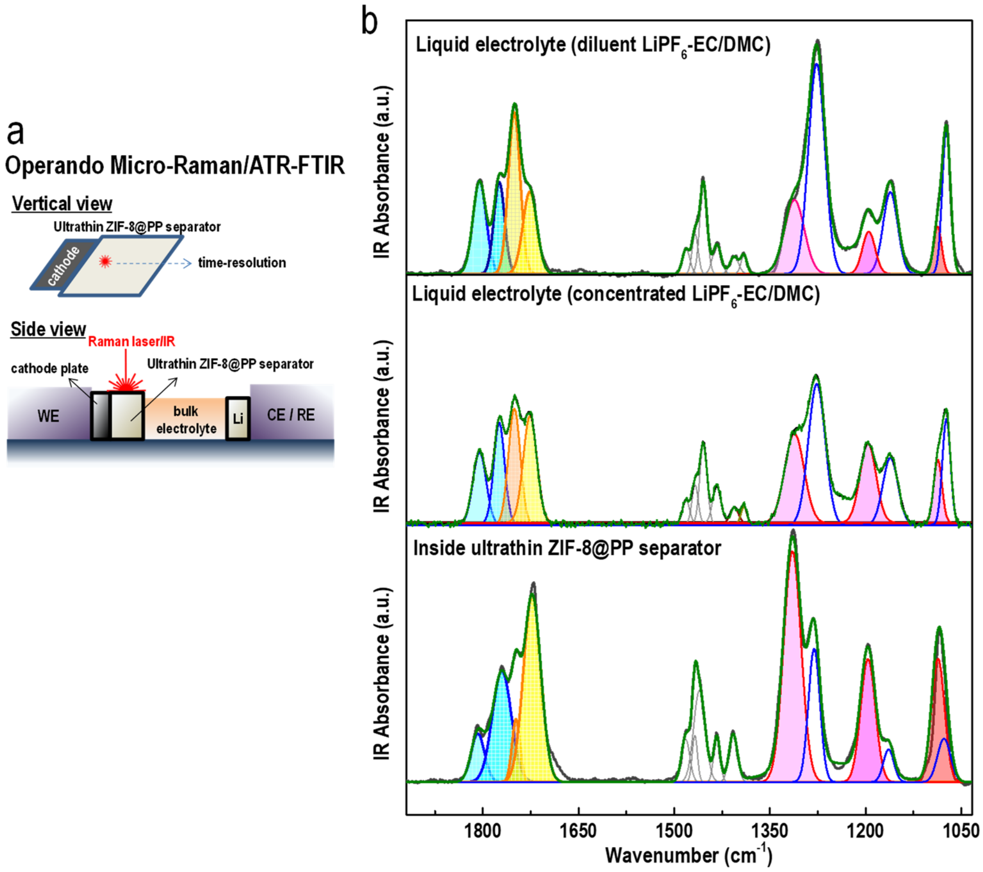

Figure S14. (a) Operando Micro-Raman and Attenuated Total Reflection–Fourier Transform Infrared (ATR-FTIR) spectra employed to characterize the configuration of typical bulk electrolyte (diluent and concentrated) and electrolyte inside improved ultrathin ZIF-8@PP separator. (b) ATR-FTIR spectra of typical diluent bulk electrolyte (upper panel), typical concentrated electrolyte (middle panel) and electrolyte inside improved ultrathin ZIF-8@PP separator (bottom panel).

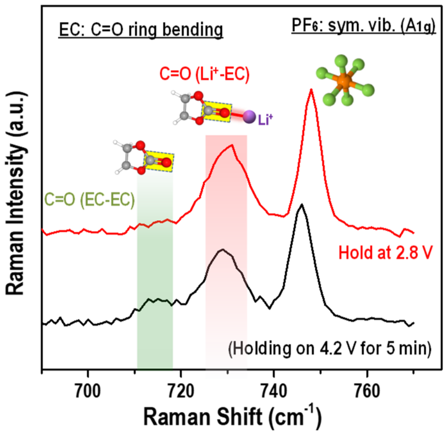

Figure S15. Raman spectra of electrolyte inside charged improved ultrathin ZIF-8@PP separator.

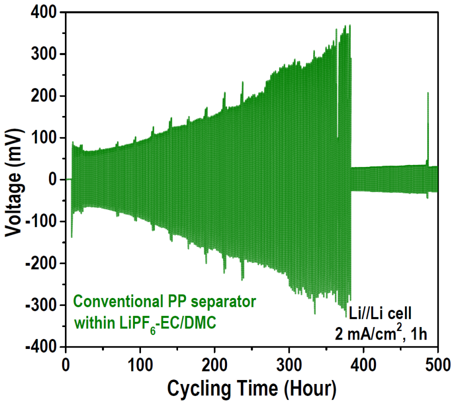

Figure S16. Li||Li symmetric cell assembled with conventional PP separator (20 μm) in typical 1 mol/L $LiPF_6$-EC-DMC carbonate-based electrolyte: 2 mA/cm^2, 1 hour.

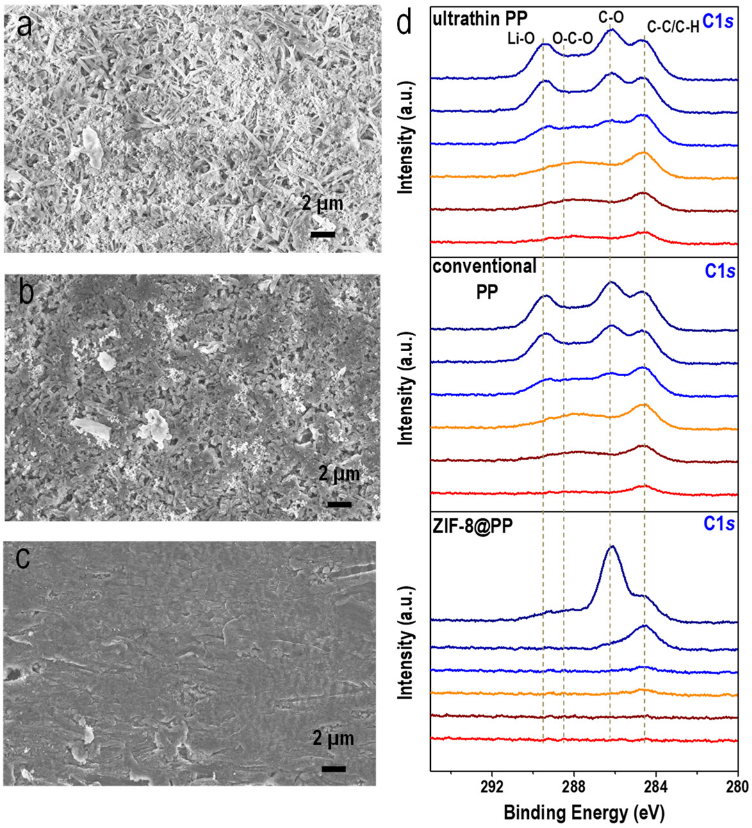

Figure S17. SEM images of the cycled lithium anodes harvested from Li||Cu half-cell (1 mol/L $LiPF_6$-EC/DMC) assembled with (a) the commercially available 8 μm ultrathin PP separator (b) conventional 20 μm PP separator and (c) the prepared improved ultrathin ZIF-8@PP separator. (d) Etching XPS spectra of C 1s recorded different time of argon ion sputtering at cycled lithium metal electrode surface of the ultrathin PP separator (upper panel), conventional PP separator (middle) and crack-free ultrathin ZIF-8@PP separator (lower panel) used Li||Cu cells, respectively.

Li anodes harvested from the cycled Li//Cu cell used prepared improved ultrathin ZIF-8@PP separator demonstrated smooth surface and without dendritic Li can be observed. In addition, remarkably suppressed electrolyte decomposition related by-products can also be found on Li anodes harvested from the cycled Li||Cu cell used prepared improved ultrathin ZIF-8@PP separator.

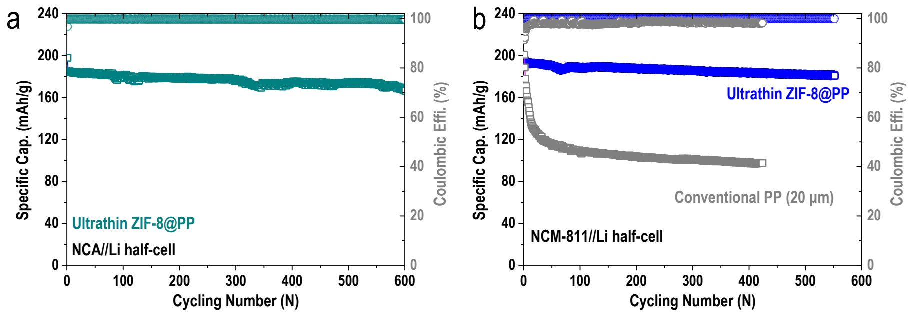

Figure S18. Cycling performances of (a) NCA||Li half-cell used improved ultrathin ZIF-8@PP separator and (b) NCM-811||Li half-cells used conventional 20 μm PP separator and improved ultrathin ZIF-8@PP separator.

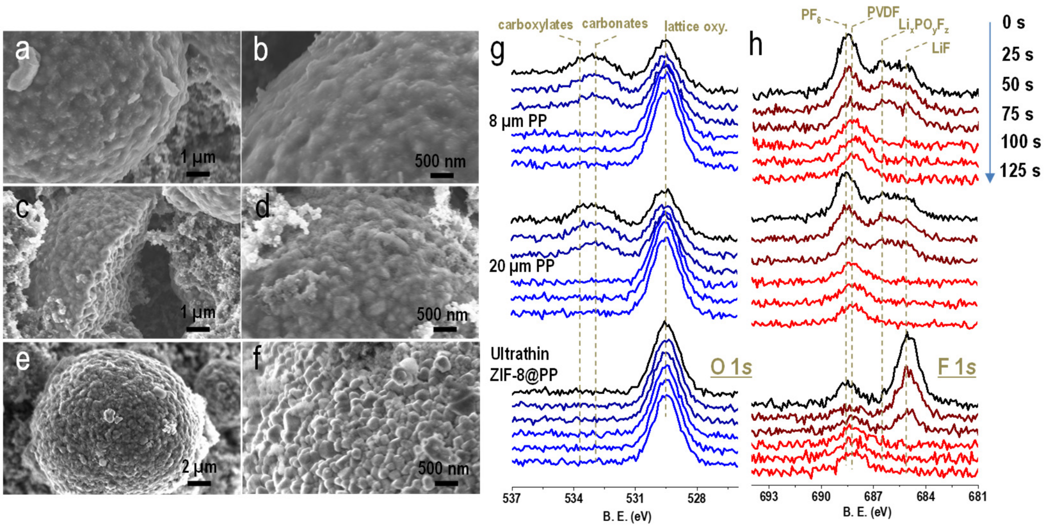

Figure S19. SEM images of the cycled NCA cathodes harvested from NCA||Li half-cell (1 mol/L $LiPF_6$-EC/DMC) assembled with (a, b) the commercially available 8 μm ultrathin PP separator (c, d) conventional 20 μm PP separator and (e, f) the prepared improved ultrathin ZIF-8@PP separator (with aggregative electrolyte confined inside). (g, h) Etching XPS results of three cycled NCA cathodes.

NCA cathodes cycled in both commercially available 8 μm ultrathin PP separator (Figure S19a, b) and conventional 20 μm PP separator (Figure S19c, d) all demonstrated obvious unevenly covered CEI layer can be distinctly observed on the surface of NCA particles. The etching XPS results shown in Figure S19g and h clearly indicated the remarkably reduced by-products as well as thin CEI layer (can be calculated by the etching time) induced by electrolyte decomposition. The thick CEI layer was caused by the serious decomposition of tremendous free-solvents that existed within the typical electrolyte's Li-ion solvation sheaths. For sharp contrast, nearly no any CEI layer can be observed on the NCA cathode surface which was assembled with the prepared improved ultrathin ZIF-8@PP separator (with aggregative electrolyte confined inside) as shown in Figure S19e and f. This completely different surface morphology can be ascribed to greatly suppressed EC/DMC solvent-related decomposition.

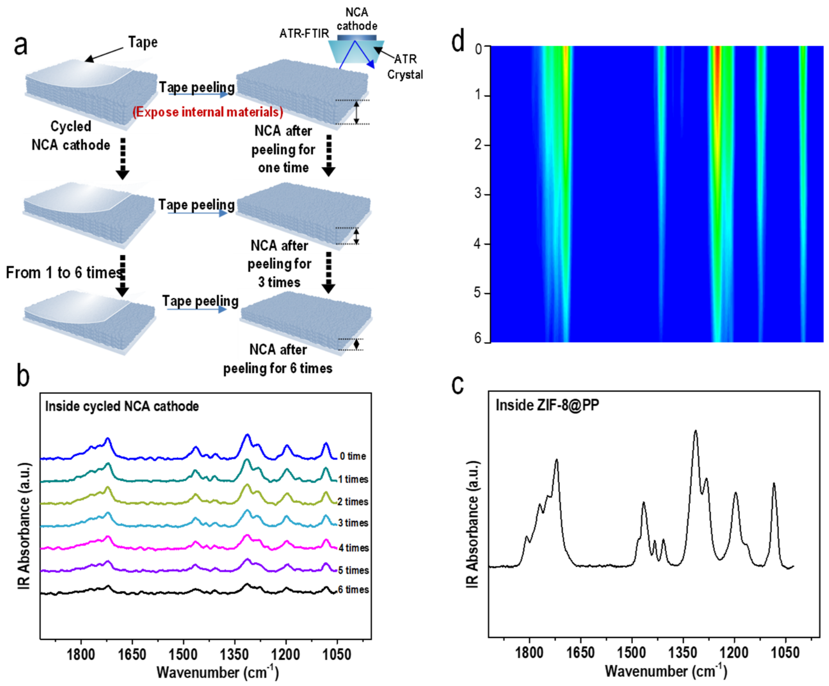

Figure S20. Tape peeling method used towards cycled NCA cathode to verify the NCA cathode was wetted by electrolyte from the ZIF-8 MOF channels. (a) Schematically illustration of the Tape peeling method employed to peel off certain amounts of NCA materials and expose the internal area of cycled NCA cathode for the following ATR-FTIR experiment. (b) ATR-FTIR spectra detected from cycled NCA cathode under different times of Tape peeling test (from 0 to 6 times) and the corresponding (d) color mapping. (c) ATR-FTIR spectrum of electrolyte within ZIF-8@PP separator.

Noting that those characterizations were only focused on the surface of NCA cathode. To further understand the working mechanism, detail information inside the deep depths of the cycled cathode needs to be further studied. Therefore, cycled NCA cathode collected from the NCA||Li used quasi-solid electrolyte was studied by ATR-FTIR. As schematically demonstrated in Figure S20a, to collect detail and accurate information even inside the deep depths of the cycled NCA cathode, a special tape peeling test was used to peel off the surface layers of the cycled NCA cathode and thus exposed the new NCA cathode interphases to the ATR-FTIR. After the different times of tap peeling (from 0 to 6 times), new interphases of NCA cathode under different depths (different thicknesses) were hence obtained. Then the obtained ATR-FTIR spectra from each depth were under further investigation. Obviously, as demonstrated in Figure S20b, several apparent peaks which were related to the liquid electrolyte, can be constantly detected under all the depths. Moreover, these peaks maintained almost the same shapes as the shapes of liquid electrolyte confined inside the MOF channels (Figure S20c). These results together suggested that the liquid electrolyte came out from the MOF channels can wet the cathode even into the deep depth and promote the lithium-ion conducting issue with the NCA cathode. despite the intensities of the electrolyte related peaks decreased gradually (Figure S20d).

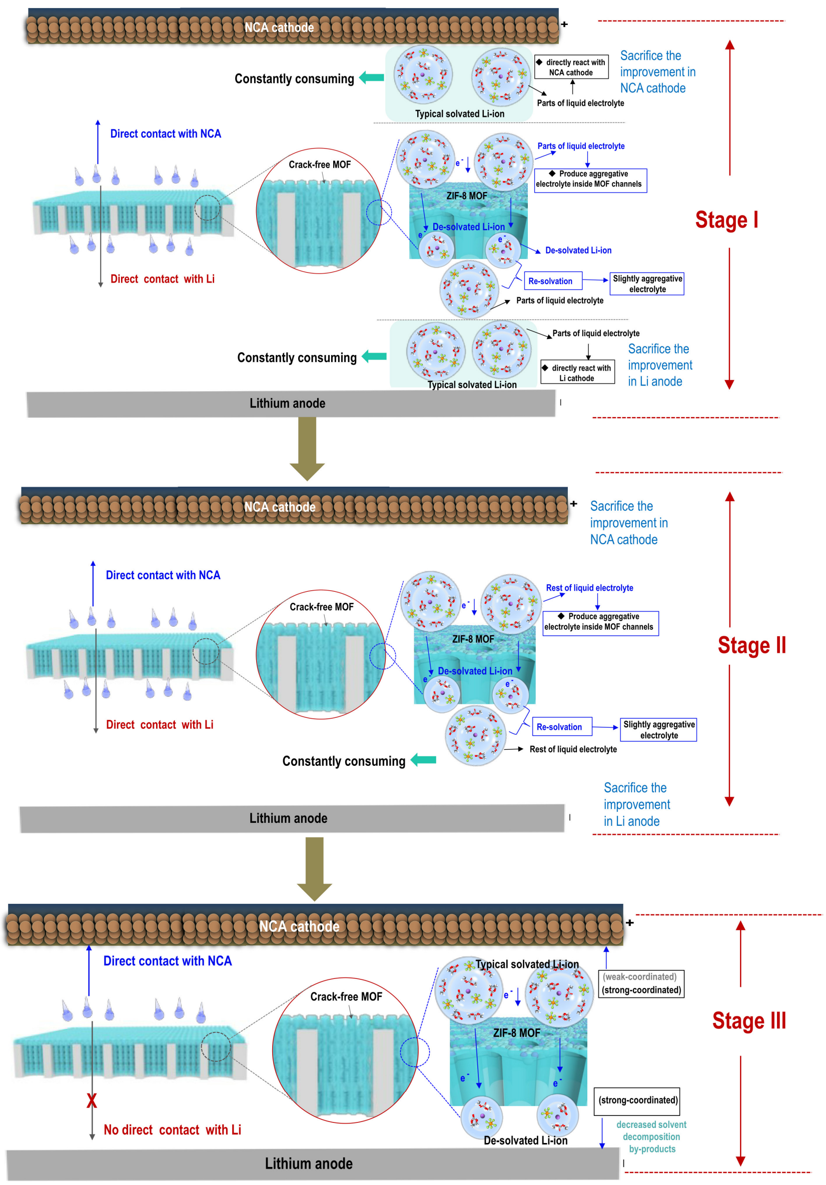

Figure S21. Schematically illustration towards the configuration evolution process of solvated lithium-ions within NCA//Li cell assembled with crack-free ultrathin ZIF-8@PP separator soaked with typical liquid electrolyte.

Actually. we think as shown in Figure S21, the formed aggregative electrolyte inside the ZIF-8 channels would re-solvated again when it went out from the ZIF-8 MOF channels. We also supposed ZIF-8@PP soaked with liquid electrolyte can be directly used and is expected to improve the electrochemical performances of batteries to some extent. Yet, the improvement may not as huge as the crack-free ultrathin ZIF-8@PP separator (with liquid electrolyte confined inside the MOF channels) can do. After the crack-free ultrathin ZIF-8@PP separator soaked with conventional liquid electrolyte, typical liquid electrolyte will exist on both sides of the crack-free ultrathin ZIF-8@PP separator. Assuming the crack-free ultrathin ZIF-8@PP separator with typical liquid electrolyte soaked is directly used to fabricate NCA//Li batteries, then during battery cycling, lithium-ions tend to experience three different processes:

namely the Stage I, Stage II and Stage III as schematically illustrated in Figure S21. Typical liquid electrolyte soaked on both sides of separator would directly contact with NCA cathode and lithium anode (the top panel of Figure S21).

During the Stage I process (at the beginning of cycling), when external electrical field is applied (battery charge/discharge), those typical liquid electrolytes may experience several different processes. From the NCA cathode side, we supposed that typical liquid electrolyte on the cathode side can be divided into two types: parts of it will directly contact with NCA cathode while the the rest parts of it will enter the MOF channels and form aggregative electrolyte (which then re-solvated with typical liquid electrolyte existed in anode side). Form the lithium anode side, there are also two types of electrolytes: parts of it will directly contact with lithium anode while the rest parts of it will re-solvated with the aggregative electrolyte formed inside the MOF channels. It worth noting that these procedures may take place simultaneously. Even parts of typical liquid electrolyte can be continuously pushed into the MOF channels and form aggregative electrolyte, however, due to the electrolyte that can enter the MOF pores is limited each time, therefore, these electrolytes that have not yet enter the MOF channels will on the one hand promote the re-solvation of aggregative electrolyte, one the other hand will react with cathode and anode. This will lead to the generation of undesirable CEI/SEI, and the growth of lithium dendrites and unstable cathode, which will ultimately affect the electrochemical performance of the NCA//Li battery.

During the Stage II process (the middle panel of Figure S21): With the deepening of the charge-discharge processes, parts of those extra typical liquid electrolyte will be constantly consumed by cathode and anode, the rest amount of typical liquid electrolyte will enter the MOF channels and form aggregative electrolyte. After that, those formed aggregative electrolyte will come out from the MOF channels and re-solvate with the rest parts of typical liquid electrolyte within the anode side, and form slightly aggregative electrolyte (compared with typical liquid electrolyte). The slightly aggregative electrolyte tends to protect the cathode and anode and consequently improve the battery performances.

During the Stage III process (the bottom panel of Figure S21): With the deepening of the charge-discharge processes, when extra typical liquid electrolyte on both the cathode and anode side is totally consumed, then the rest part of typical liquid electrolyte will enter the MOF channels and form aggregative electrolyte without further experience re-solvation process. Noting that the Stage III process is actually the core innovation of this work as shown in Figure 4a in our manuscript. Therefore, both the NCA cathode and the lithium anode will free from EC/DMC solvent-related decomposition, and the corresponding electrochemical performance will be improved.

Therefore, ZIF-8@PP soaked with liquid electrolyte can be directly used and improve the electrochemical performances of batteries to some extent. Yet, the improvement may not as huge as the crack-free ultrathin ZIF-8@PP separator (with liquid electrolyte confined inside the MOF channels) can do. This further suggests the importance of using improved ultrathin ZIF-8@PP separator with aggregative electrolyte inside MOF channels to fabricate various batteries with excellent performances.

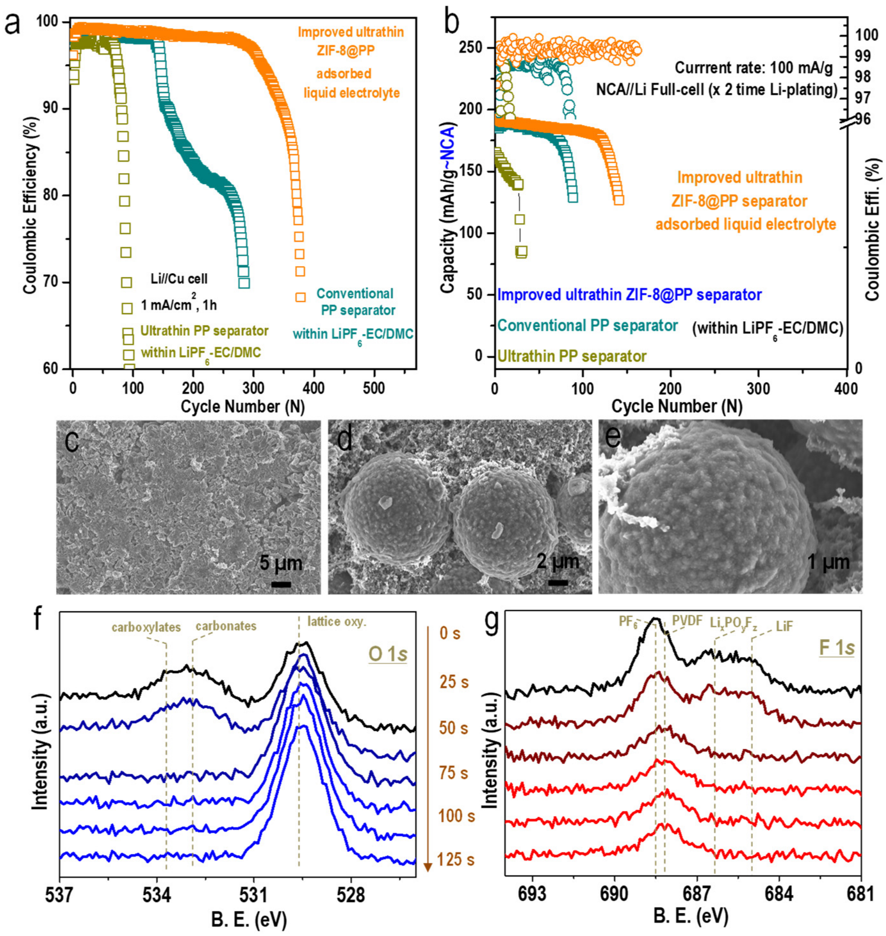

Figure S22. Electrochemical performances of (a) Li||Cu half-cell and (b) NCA||Li full-cell assembled with crack-free ultrathin ZIF-8@PP separator soaked with typical liquid electrolyte. SEM images of (c, d) cycled NCA cathode and (e) cycled lithium anode harvested from (b). And the corresponding (f) O1s and (g) F1s XPS spectra of the cycled NCA.

The data related to ultrathin PP (dark yellow curves) and conventional PP separators (dark green curves) in Figure S22 were re-draw from Figure 5b and 5d in manuscript.

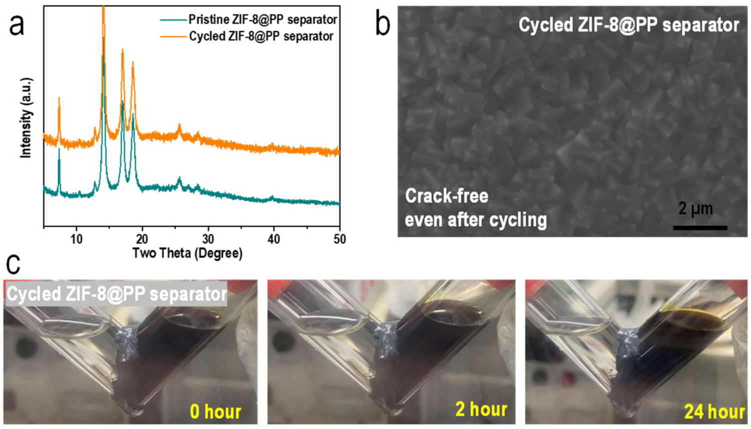

Figure S23. Characterizations to verify the crack-free property of the cycled improved ultrathin ZIF-8@PP separator. (a) XRD pattern of the pristine and cycled improved ultrathin ZIF-8@PP separator. (b) SEM image of the cycled improved ultrathin ZIF-8@PP separator. (c) Polysulfide diffusion experiment by using the cycled improved ultrathin ZIF-8@PP separator.

The XRD of the cycled improved ultrathin ZIF-8@PP separator was almost the same as that of the pristine improved ultrathin ZIF-8@PP separator, which suggested the excellent stability of the cycled improved ultrathin ZIF-8@PP separator. The SEM shown in Figure S23b also indicated the cycled improved ultrathin ZIF-8@PP separator can still preserve its original crack-free structure. Moreover, when used in polysulfides diffusion experiment, no polysulfides can diffusion across the cycled improved ultrathin ZIF-8@PP separator, which further suggested the crack-free structure.

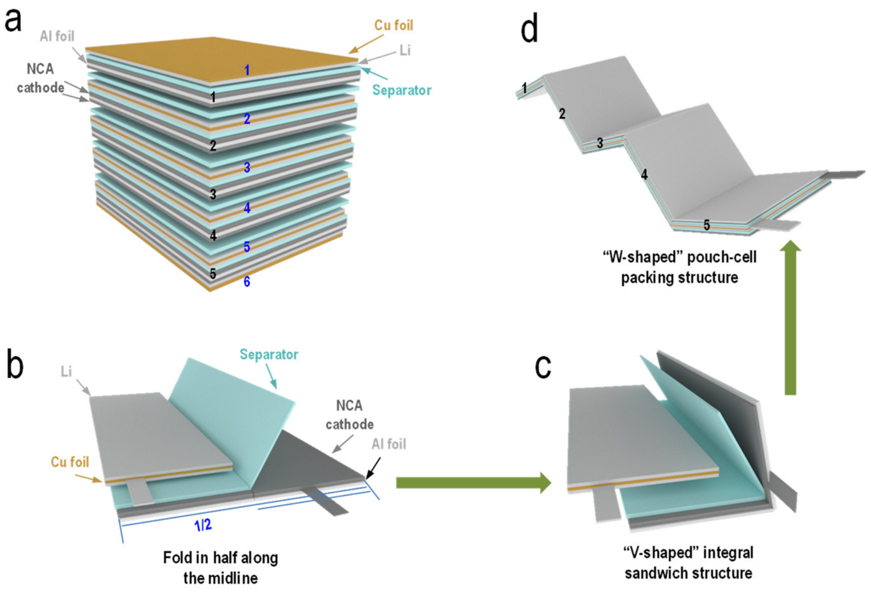

Figure S24. Packing structure of pouch-cell fabricated by (a) conventional method and (b-d) "W-shaped" method in this work.

For most pouch-cell fabrications, there were one more layer of anode layers than that of the cathode layers (as schematically illustrated in Figure S24a). However, the way we fabricated the pouch-cell was different from that generally adopted. As shown in Figure S24b, the size of the anode side is half of that of the cathode side and ZIF-8@PP separator. The anode with lithium metal coated on both sides of Cu foil is sandwiched between the 1/2-folded separator and cathode (Figure S24b, NCA coated on only one side of the Al foil). Then, a "V-shaped" integral sandwich structure was obtained (as schematically illustrated in Figure S24c, both the NCA cathode and the ZIF-8@PP separator are folded in half along the direction parallel to the tabs, the lithium anode was sandwiched between the folded cathode & ZIF-8@PP separator). Then, the whole integral sandwich structure was folded four times and formed a "W-shape" pouch-cell packing structure (Figure S24d). Hence, only used 5 layers of lithium anodes in this work.

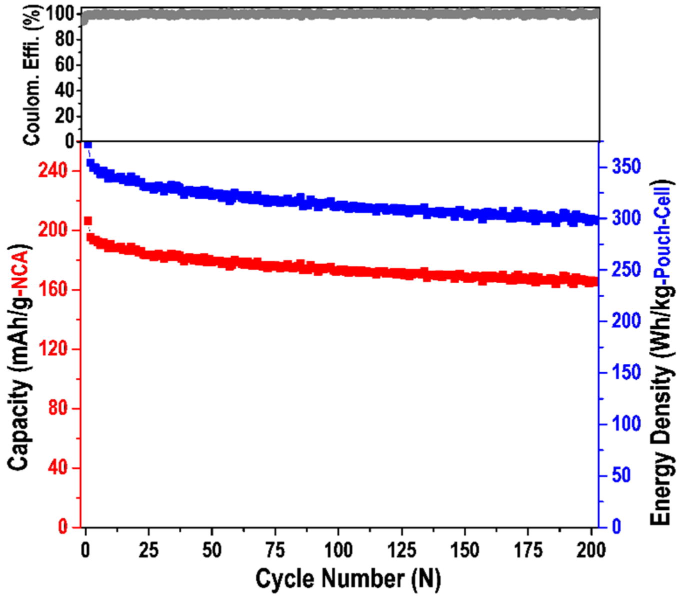

Figure S25. Specific capacity and energy density of the 350 Wh/kg NCA||Li pouch-cell assembled with the improved ultrathin ZIF-8@PP separator in typical 1 mol/L $LiPF_6$-EC/DMC carbonate-based electrolyte.

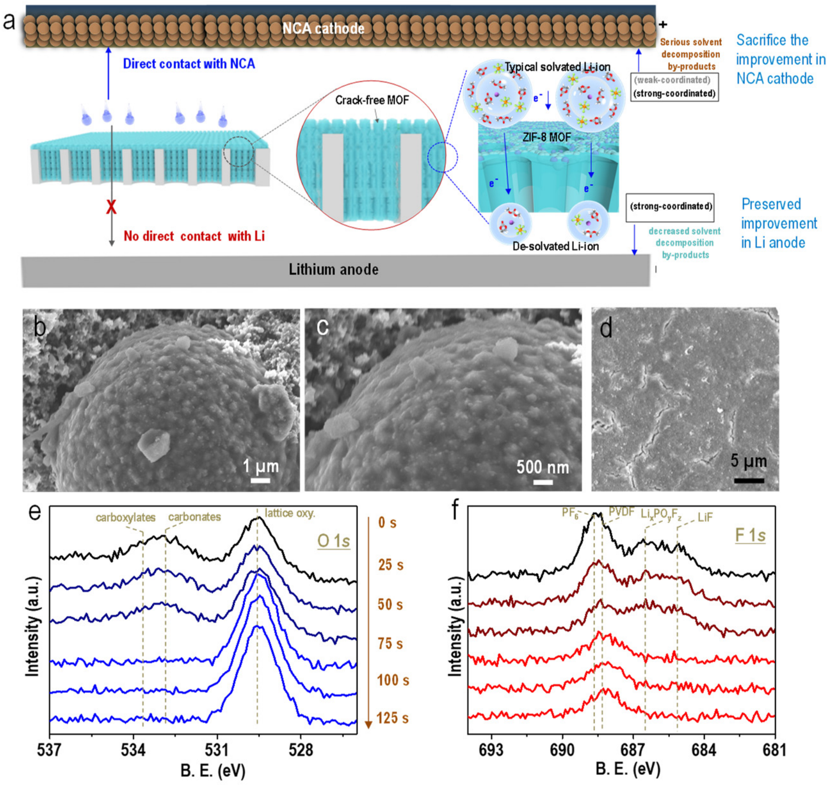

Figure S26. Schematically illustration of the assembling process of NCA//Li pouch-cell assembled with crack-free ultrathin ZIF-8@PP separator and extra amount of liquid electrolyte added. SEM image and the corresponding XPS spectra of the O1*s* and F1*s* of cycled NCA cathode from NCA//Li pouch-cell assembled with crack-free ultrathin ZIF-8@PP separator. SEM image of cycled lithium from NCA//Li pouch-cell assembled with crack-free ultrathin ZIF-8@PP separator.

In fact, during the assembling of pouch-cell, the extra amount of liquid electrolyte was directly added on one side of the crack-free ultrathin ZIF-8@PP separator which facing the NCA cathode (as schematically illustrated in Figure S26a). Thus, additional added liquid electrolyte was not directly contact with lithium anode, which means solvated lithium-ions within the additional added liquid electrolyte still need to drop off parts of their solvation solvents and transport through the MOF channels before reach the lithium anode. Therefore, liquid electrolyte finally contact with lithium anode was still possess aggregative configuration. For this reason, the crack-free ultrathin ZIF-8@PP separator with aggregative electrolyte confined inside MOF channels can still stabilize lithium anode even after additional liquid electrolyte was added into pouch-cell. However, the additional liquid electrolyte added in the cathode side may sacrifice the improvement in NCA cathode previously brought by the aggregative electrolyte. However, since the lithium anode is more vulnerable in carbonated-based electrolyte (1M $LiPF_6$-EC/DMC electrolyte) than the NCA cathode, after carefully considering the pros and cons (prioritizing to stabilize the more vulnerable lithium anode and sacrifice the possible further enhancement on NCA cathode), we decided to add the additional

liquid electrolyte on the surface of crack-free ultrathin ZIF-8@PP separator facing the NCA cathode side.

To further support our conjecture/explanation, we also supplemented the corresponding SEM and XPS data of the cycled NCA cathode and lithium anode harvested from NCA//Li pouch-cell. Clearly, as shown in Figure S26d, the cycled lithium anode exhibited a flat and dendrite-free surface similar as that harvested from NCA//Li coin-cell used crack-free ultrathin ZIF-8@PP separator (with aggregative electrolyte confined inside the MOF channels) as shown in Figure 5b in our original manuscript. For the cycled NCA cathode, the morphology and surface CEI information were similar as that of the NCA cathode harvested from NCA//Li coin-cell used typical liquid electrolyte and conventional separator (as shown in Figure S19). Therefore, the crack-free ultrathin ZIF-8@PP separator (with aggregative electrolyte confined inside MOF channels) can still stabilize lithium anode even after additional liquid electrolyte was added into pouch-cell.

Table S1. Details of pouch cell parameters demonstrated in Figure 6.

Parameter	Pouch Cell
Specific Capacity (NCA)	196.4 mAh/g
Mass Loading (NCA)	25.2 ± 0.2 mg/cm^2
Cathode Mass Loading	31.5 ± 0.25 mg/cm^2 (NCA: 80 wt%)
Area Capacity (single face)	4.95 ± 0.4 mAh/cm^2
Ave. Out-put voltage	3.76 V
Active Area	(4 x 5) x 2 x 5 cm^2 = 200 cm^2
Al foil (10 µm)	0.309 ± 0.02 g
Li anode (100 µm)	1.068 ± 0.04 g
Cu foil (9 µm)	0.746 ± 0.02 g
sN/P ratio	3.96
Electrolyte	0.900 g
Separator (Ultrathin ZIF-8@PP)	0.103 g
Package + Tabs	1.08 g
Pouch-Cell Weight	10.506 g
Capacity	0.99 Ah
Energy	3.72 Wh
Energy Density (weight)	354.3 Wh/kg

Supplementary References

1. Zhao, Y. et al. Flexible Polypropylene-Supported ZIF-8 Membranes for Highly Efficient Propene/Propane Separation. *J. Am. Chem. Soc.* **142**, 20915-20919 (2020).
